# Research on mechanical properties and prediction methods of hybrid fiber concrete for airport pavements

**DOI:** 10.1371/journal.pone.0331951

**Published:** 2025-11-11

**Authors:** Wenyuan Xu, Zhen Zang, Xiu Liu, Yongcheng Ji, Jianbo Xu, Hao Li

**Affiliations:** School of Civil Engineering and Transportation, Northeast Forestry University, Harbin, Heilongjiang Province, China; Shandong University of Technology, CHINA

## Abstract

The cement concrete pavement at airports is prone to fatigue cracking due to dynamic loads from airplanes and environmental coupling effects. This research developed a strength prediction model and introduced a novel hybrid fiber effect coefficient (RH) by conducting both single and mixed experiments with polypropylene (PP), polyvinyl alcohol (PVA), basalt (BF), and polyacrylonitrile (PAN) fibers, alongside mechanical testing and significance analysis. All three hybrid fibers exhibited favorable hybrid effects, with optimal dosages as follows: compressive strength for PPFRC (0.38, 0.11), BFRC (0.6, 0.4), PNCC (0.5, 0.1); and flexural strength for PPFRC (0.5, 0.1), BFRC (0.6, 0.03), PNCC (0.4, 0.1), respectively. This accomplishment can be utilized in future airport engineering, offering distinct alternatives for runways (ideally BFRC) and aprons (preferably PPFRC/PNCC), substantially prolonging road surface lifespan to 20 years and diminishing comprehensive maintenance expenses.

## Introduction

Cement concrete is a composite material, and the majority of civil airports are constructed with cement concrete pavements. The prolonged coupling of aircraft dynamic loads with severe environmental conditions leads to shrinkage, cracking, and fatigue deterioration, which significantly jeopardizes the longevity and safety of the pavement [1–3]. Fiber-reinforced concrete (HFRC), a composite material [4], has anti-cracking and toughening properties due to the incorporation of concrete with different fibers. Historically, the primary fibers utilized by researchers to augment the strength of concrete are basalt fibers [5,6] and synthetic fibers [7,8]. The incorporation of a singular fiber can only enhance performance from a specific perspective [9–11], making it challenging to simultaneously improve multiple attributes [12,13]. The amalgamation of fiber with varying qualities and dimensions can optimise the scale and performance characteristics of diverse fiber in concrete, so harnessing their strength at multiple scales and performance levels to generate a synergistic impact and increase the overall outcome. Mixed fibre concrete employs diverse fibre dimensions to create graded synergistic mixing effects, enhancing toughness throughout various loading stages and structural levels.

Currently, research by domestic and international scholars on HFRC primarily focuses on its fundamental mechanical properties [14,15], while the synergistic mechanism of its hybrid effect remains ambiguous, and there is an absence of systematic analysis regarding the synergistic mechanism of hybrid fibers within multi-scale structures. Airport pavements differ from conventional cement concrete pavements as they must endure high-frequency impacts and repetitive pressures during aircraft takeoff and landing, necessitating great performance, extended service life, and little maintenance. The demand for hybrid fiber is propelled by their superior strength, fatigue resistance, cost efficiency, and particular material specifications, as evidenced by projects like Beijing Daxing International Airport, Chengdu Tianfu International Airport (TFU), and Kunming Changshui International Airport (KMG). These projects utilise a combination of several forms of fiber-reinforced concrete (steel fiber, synthetic fiber, basalt fiber) for the design of taxiways, aprons, and similar structures. In comparison to conventional concrete, their mechanical qualities, durability, and strength are markedly enhanced. The mechanical characteristics, durability, and strength of concrete are markedly enhanced, consequently diminishing maintenance expenses during its entire duration. Following two years of operational monitoring, the occurrence of pavement cracks in associated projects has diminished by over 70% relative to conventional concrete, and maintenance expenditures have decreased by more than 40%. While conventional experimental techniques yield precise results, they exhibit drawbacks related to cost and time efficiency, and struggle to thoroughly encompass diverse combinations of material ratios [16,17]. Consequently, the advancement of efficient predictive methodologies is crucial for optimizing the design of hybrid fiber composites, and the identification of hybrid fiber types appropriate for airport pavements is the subsequent phase of this research. This paper’s study focus will address the knowledge gap regarding hybrid fibers throughout the design-performance-implementation continuum in airport engineering, offering theoretical support for durable and low-maintenance paving materials.

To resolve the aforementioned concerns, a selection of fibers was chosen for this investigation. The materials comprised synthetic coarse polypropylene fibers (PP), polyvinyl alcohol (PVA) synthetic fibers, modified impregnated basalt fibers (BF), and polyacrylonitrile (PAN) as fine fibers. Polypropylene polyvinyl alcohol fiber-reinforced concrete (PPFRC), polypropylene basalt fiber-reinforced concrete (BFRC), and polypropylene polyacrylonitrile fiber-reinforced concrete (PNCC) were made from standard concrete (RC). The impact of single-fiber reinforced concrete and various hybridfiber types on the mechanical characteristics of pavement was examined, and predictive models for the mechanical properties of both single-fiber and mixed-fiber concrete were developed. A new mixed-fiber effect coefficient, $R_{H}$was established. It was a new method for quantifying synergistic effects.

## Raw material and mixing design

### Raw materials

Cement: M32.5 composite silicate cement

Aggregate: Crushed limestone aggregate. Two grading categories: 5–10 mm and 10–20 mm, with a grading ratio of 3:7. Fine aggregate consists of river sand.

This study utilized a high-performance water-reducing chemical composed of polycarboxylic acid. An antifoam chemical was utilized at a concentration of 0.1% of the cement mass to reduce the production of air bubbles caused by fiber inclusion.

**Fig 1:**
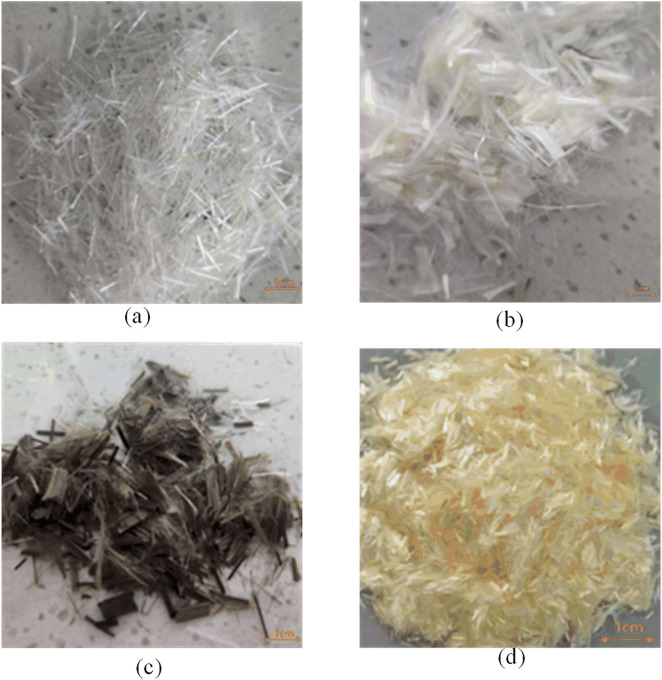
Schematic of fiber appearance.

Four unique fiber types—PP, PVA, BF, and PAN—were chosen for inclusion in a specified percentage within the study. The specific performance indices related to each fiber are displayed in Table 1. The diameter and length of the fibers were diminished to examine the influence of various fine fiber types on the performance of hybrid fiber concrete. This was executed to guarantee uniformity in the fibers’ appearance, as illustrated in Fig 1. Three categories of fine fibers were chosen for the studies, differing in diameter and length, to investigate their impact on strength. The choice of fibre manufacturer brands guarantees alignment with pertinent airport infrastructure initiatives.

**Table 1:** Fiber material parameters.

Fiber type	Diameter/μm	Tensile strength /MPa	Tensile limit /%	Length /mm	Density (kg/m³)	Modulus of elasticity (Gpa)	geometry
PP	750	700	6	25	7.9	10	hooked
PVA	15 ~ 48	500	15	6	1.3	4.8	fasciculated
BF	7 ~ 25	1050	12	6	0.91	35	fasciculated
PAN	13 ~ 25	600	15	6	1.18	7.18	spiral

The experimental water was potable.

### Hybrid design

According to the design parameters, experience, and pertinent research [18–21], the reference concrete mix ratio for 1 m³ of concrete consists of 331 kg of cement, 136 kg of water, 623 kg of sand, and 1346 kg of stone (refer to Table 2).

**Table 2:** Concrete reference ratios.

Consumption of concrete per square meter (kg)	Clinker	Water	Granule	Boulder	Small stone
	331	136	623	943	403

### Mixing technology

**Fig 2:**
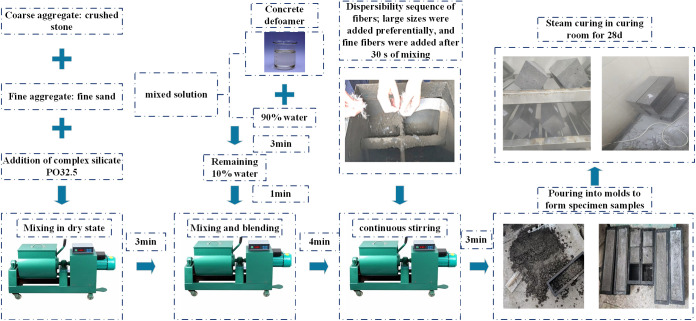
Flow chart of sample preparation.

The preparation of hybrid fiber concrete using various fibers is a complex and essential operation. The aggregation of concrete fibers results in a significant quantity of air bubbles. These bubbles serve as stress concentration areas during mechanical property testing, therefore diminishing the mechanical qualities of the concrete. The even distribution of fibers inside the composite is essential for achieving good strength and toughness properties. A significant amount of research has focused on fiber dispersion, with prior scholars doing thorough investigations on this topic [22–24]. Consequently, it is essential to guarantee that the fibers are completely dispersed during the mixing procedure. The procedure is illustrated in Fig 2.

## Strength characterization

### Specimen design and fabrication

**Fig 3:**
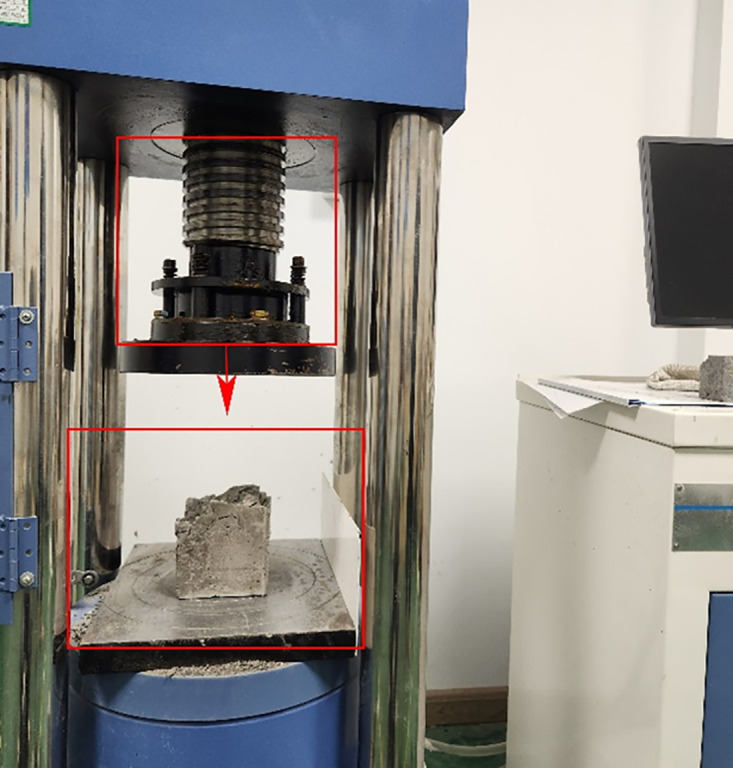
Schematic diagram of compressive strength test and damage location of cubic specimen (100 mm × 100 mm × 100 mm).

The Standard of Test Methods for Fiber Concrete specifies that the flexural and compressive strength of concrete be assessed through a series of rigorous tests. The dimensions of the flexural specimens are 100 millimeters by 100 millimeters by 400 millimeters, with three specimens in each group. The compressive strength is assessed after the flexural specimen, which measures 100 millimeters by 100 millimeters by 100 millimeters, with three specimens per group. Four unique fiber combinations were chosen for the hybrid formulation of PPFRC, BFRC, and PNCC: PP, PVA, BF, and PAN. The cube compressive strength test and on-site testing are shown in Fig 3, and the bending toughness loading device and on-site testing are shown in Fig 4.

### Loading device

**Fig 4:**
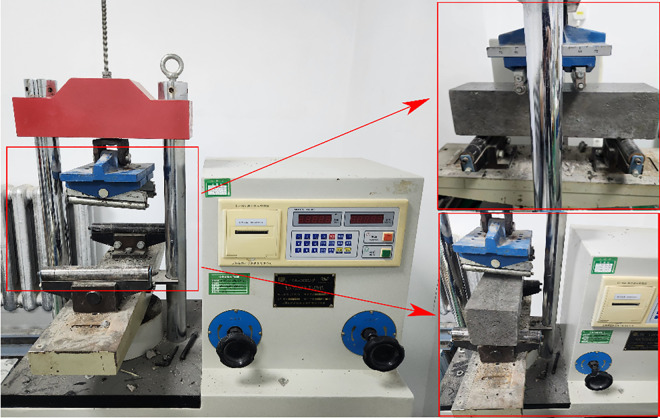
Prism specimen (100 mm × 100 mm × 400 mm), three-point bending toughness testing device, and on-site diagram.

## Test results and analysis

### The strength of concrete that has been reinforced with a single fiber

**Fig 5:**
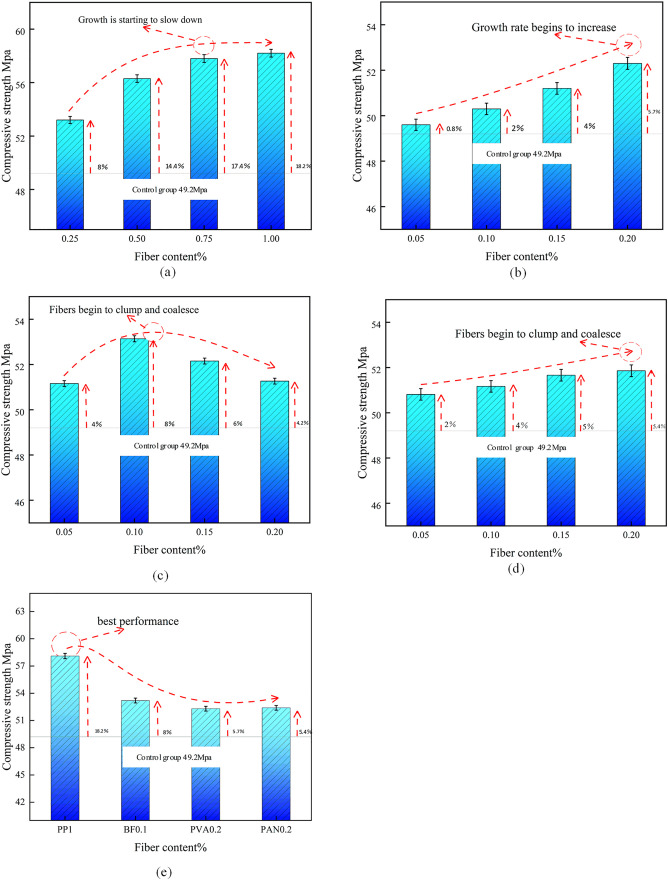
Role of various types of fibers in increasing the compressive strength of concrete.

To enable a comparative analysis with the outcomes of the hybrid fiber test, four fiber types were individually mixed for compressive and flexural testing. The results of these tests are illustrated in Fig 5.

#### Comparative analysis of compressive strength.

Fig 5 illustrates the impact of various fiber types on the compressive strength of concrete with distinct fiber admixtures. The graphic illustrates that all fiber types can enhance the compressive strength of the substrate to a certain degree. The efficacy of improvement based on fiber type is ranked as follows: PP, BF, PVA, PAN. Polypropylene (PP) exhibits the most significant enhancement in strength, primarily due to its superior tensile strength, modulus of elasticity, and end anchoring effect. Additionally, the PP fibers possess a substantial aspect ratio, resulting in minimal impact on the fluidity of the substrate pulp. However, as the content of PP fibers increases, a decline in compressive strength is observed, with a mere 1% increase in strength at 1.0% fiber content, suggesting that fiber agglomeration may commence with higher fiber concentrations. The optimal basalt fiber content for achieving the best lifting effect is 0.1%. At this level, it provides a reinforcing effect; however, as the fiber content increases, the strength begins to diminish. This decline is attributed to the smooth surface of the basalt fiber, which reduces the frictional resistance during pulling, leading to agglomeration and consequently a reduction in strength. Enhancement effects of PVA and PAN fibers. The reinforcement effects of PVA fiber and PAN fiber are comparable, as their fiber length, diameter, and modulus of elasticity exhibit minimal differences. The efficacy of these two synthetic fiber-reinforced concrete types arises from their excellent adhesion to the substrate and closely spaced fibers, which, during the concrete molding and curing process, effectively fill the aggregate spaces, resulting in a dense structure that enhances strength. It is important to acknowledge that PVA fiber, owing to its fine characteristics and water absorption properties, exhibited agglomeration during testing. Therefore, careful consideration must be given to the quantity of PVA fiber, as an increase in material will influence the fluidity and strength of the paste. The length and fineness of PAN fiber are relatively inferior to those of PVA fiber, resulting in a less pronounced impact compared to PVA fiber.

#### Comparative analysis of flexural strength.

**Fig 6:**
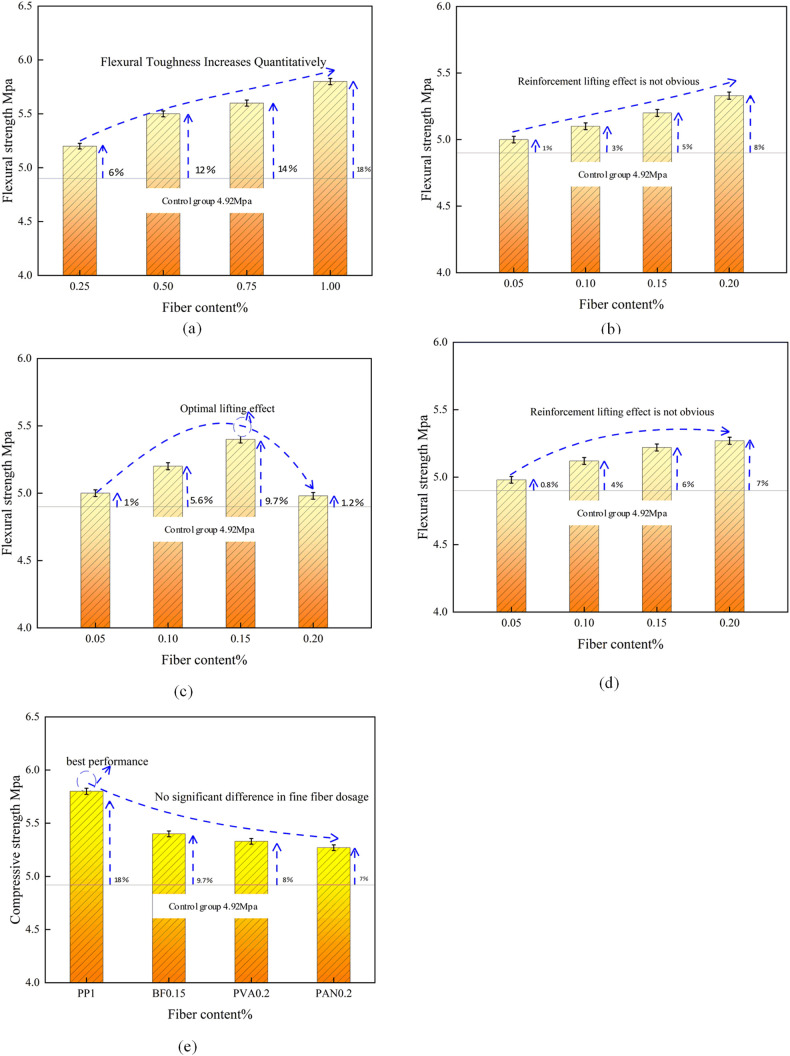
Curve of flexural strength of single fiber concrete (28-day curing) with fiber content variation.

Fig 6 displays the flexural strength values of various fiber-reinforced concrete specimens.

Fig 6 illustrates that, akin to the compressive strength results, all fibers enhanced the flexural strength of the substrate to varying degrees. The hierarchy of effects, from greatest to least, is as follows: PP > PVA ≈ PAN > BF. Among the fibers, PP fiber demonstrates the most significant enhancement impact; an increase in fiber content correlates with improved flexural performance, and the growth rate exhibits a consistent upward trend. Upon reaching a fiber content of 1%, the bending strength begins to grow markedly. The performance enhancement of PVA fiber and PAN fiber is comparable; nevertheless, the highest performance improvements are merely 8% and 7%, respectively. The enhancement of these two synthetic fine fibers parallels that of compressive strength; thus, their impact on the flexural strength of concrete is negligible. The interface of BF fibers is excessively smooth; nonetheless, the dispersion of high-density fibers imparts a degree of flexural capability. Nevertheless, the flexural strength declined significantly when the fiber content exceeded 1.5%, mirroring the findings of the compressive strength test. The optimal content of BF fiber for enhancing flexural strength is between 0.1% and 0.15%, indicating that BF fiber has a negligible effect on the flexural strength of concrete.

In conclusion, the ideal fiber content is 1% for polypropylene (PP) fibers and 0.15% for basalt fibers (BF). When comparing fiber contents of 0.1% and 0.15%, the compressive strength increases by 8% and 6%, respectively, with negligible differences. However, the flexural strength improves by 9.7% at 0.15% content, resulting in superior pulp molding effects and enhanced workability with basalt fibers at this concentration. The ideal concentration of PVA and PAN fibers should range from 0.15% to 0.2% or even exceed this range. In selecting the mixing ratio, it is advisable to use fibers at the lower limit of the ideal fiber content, since the synergistic effects of the four types of fibers are promising.

### Strength of concrete reinforced with hybrid fibers

The compressive and flexural strengths of the hybrid fiber concrete specimens, both before and following the incorporation of fibers, were evaluated, and their damage patterns were examined. The findings from the single-fiber reinforced concrete strength test and prior studies [25,26]. Indicate that the optimal mixing ratio of mixed fine fibers should range from 0.05% to 0.2%, aligning with the requirements of the actual airport pavement project. To investigate the synergistic effects of hybrid fibers while minimizing potential multivariate errors in testing, the variable is constrained to 0.5% of the volume fraction of crude fibers (PP). 0.5%, analyzing various fine fiber kinds, the influence of fine fiber volume fraction on the mechanical properties of high-performance concrete. Establishing the test variable components as delineated in Table 3, together with the test program and specimen categorization as presented in Table 4.

**Table 3:** Experimental variable factors.

Variant	Considerations	
	Fine fiber types	Fine fiber volume fraction/ %
1	PVA	0.05
2	BF	0.1
3	PAN	0.15

**Table 4:** Experimental program and test groups.

Serial number	Clusters	Fine Fiber Types	Crude Fiber Volume Fraction%	Fine fiber volume fraction/%
N0	PC Group	**—**	**—**	**—**
N1	PPFRC Group	PVA	0.5	0.05
N2		PVA	0.5	0.1
N3		PVA	0.5	0.15
N4	BFRC Group	BF	0.5	0.05
N5		BF	0.5	0.1
N6		BF	0.5	0.15
N7	PNCC Group	PAN	0.5	0.05
N8		PAN	0.5	0.1
N9		PAN	0.5	0.15

#### Experimental phenomena.

(1) Analysis of test phenomena of compressive specimens

**Fig 7:**
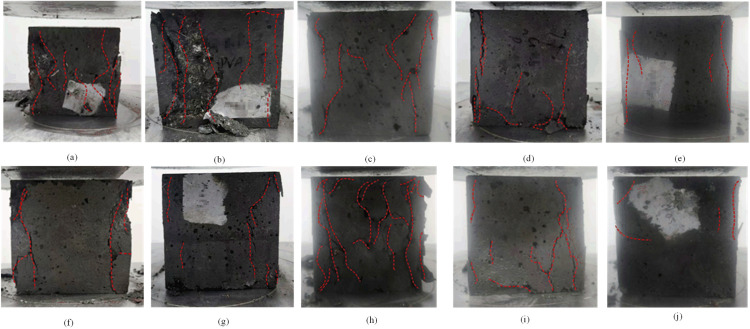
Compressive damage patterns of hybrid fiber concrete and ordinary concrete specimens. **(a)** Plain concrete (N0); **(b-j)** Hybrid fiber group: N1-N9 (see Table 4 for fiber ratio).

Fig 7(a) illustrates that upon loading the plain concrete (P-C) specimens, an increase in stress led to rapid damage after reaching peak stress. The concrete surface, perpendicular to the bearing surface, disintegrated and detached, accompanied by the audible sound of compression cracking, as depicted in Fig 7(a). In comparison to plain concrete, hybrid fiber concrete specimens exhibit a greater number of cracks and surface delamination after attaining peak stress. As the loading process persists, the cracks progressively lengthen and widen, predominantly concentrating on the specimen’s exterior. As the total fiber content rises, the shedding of surface cracks diminishes. Surface cracking and shedding are particularly pronounced in groups N1, N7, and BFRC concrete examples, as illustrated in Fig 7e-7g. In comparison to PPFRC, the surface shedding fragments of PNCC are smaller, and the cracks are more delicate. The overall integrity of the three mixed-fiber concretes surpassed that of the plain concrete specimens, exhibiting prolonged damage duration.

(2) Analysis of test phenomena of flexural specimens

**Fig 8:**
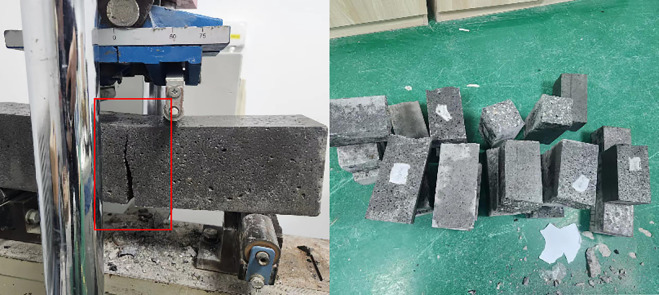
Flexural toughness damage pattern.

The bending toughness damage mode is shown in Fig 8. All specimens displayed brittle fracture without previous warning; the first crack simultaneously initiated at three equidistant spots on the tensile surface, swiftly propagating upward to form a flat region throughout the whole cross-section. This was followed by a rupture, resulting in an immediate bifurcation, exposing filamentous threads inside the cross-section. The standard fiber specimen (PC group) had damage marked by a solitary vertical fissure at the base of the central section. The composite fiber specimen exhibited a more significant bending deformation in the compression zone; as fiber doping increased, the rate of primary crack propagation slowed, but bifurcation did not take place. The fracture surface exhibited discernible evidence of fiber bridging in some regions. The results demonstrate that fiber doping slightly postponed crack advancement; however, insufficient bonding at the fiber-matrix interface and the lack of a multi-scale fiber synergistic effect impeded the effective activation of the multi-crack ductility mechanism, leading to a sudden single-crack fracture as the primary failure mode.

(3) Analysis of Microscopic Phenomena

**Fig 9:**
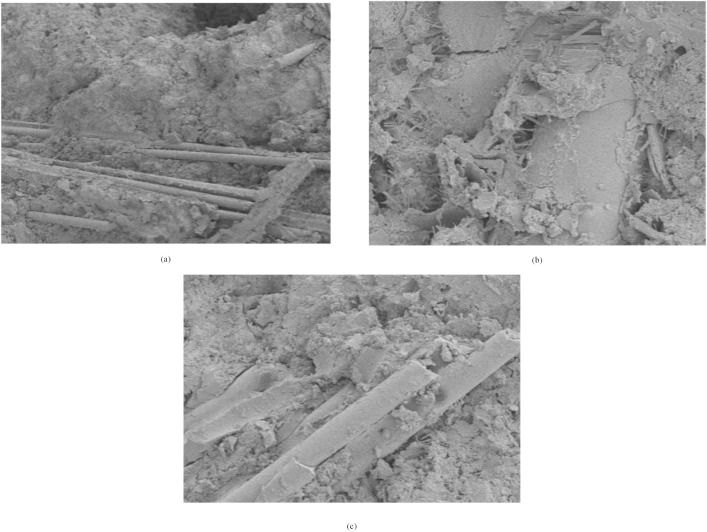
HFRC SEM image.

The microstructure of three varieties of hybrid fibre reinforced concrete (PPFRC, BFRC, PNCC) was examined using scanning electron microscopy (SEM) (Fig 9), elucidating the properties of the fiber-matrix interface and the hybrid reinforcing mechanism. The substantial hydration

products adhering to the surface of PVA fibres in PPFRC demonstrate its hydrophilicity and robust chemical interaction with cement slurry. The polypropylene coarse fibres and polyvinyl alcohol fine fibres create a multi-tiered network that spans macroscopic fissures, while the fine polyvinyl alcohol fibres occupy microcracks to inhibit crack advancement. The absence of debonding at the interface between the two confirms optimal flexural strength; the smooth and inert surface of BF fibres in BFRC results in pores inside the interface transition zone. Nonetheless, the termini of PP coarse fibres interlace with BF fibres to create a physical interlocking configuration, compensating for the inadequate bonding at the BF interface; the helical structure of PAN fibres in PNCC amplifies their mechanical anchoring effect with the matrix, leading to localised fibre aggregation. Nonetheless, PP fibres create a “skeleton support” encircling the PAN aggregates, mitigating the effects of flaws.

Of the three categories of mixed fibres, coarse fibres (PP) mitigate macroscopic cracks, whilst fine fibres (PVA/BF/PAN) alleviate microscopic damage, hence attaining multi-level crack resistance and synergistic effects across many scales.

#### Test results.

**Fig 10:**
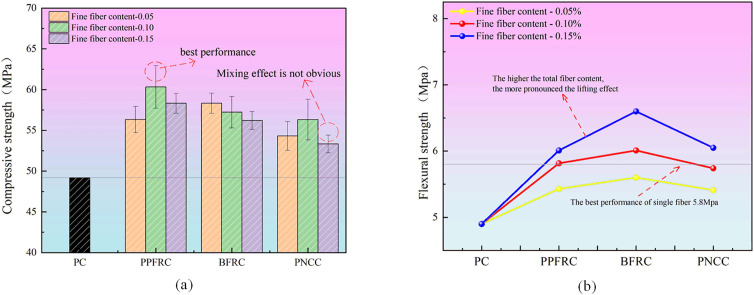
Plot of HFRC mechanical property data.

Fig 10 displays the outcomes of the strength tests on the test data.

(1) Effect of fiber variety on strength

Fig 6 illustrates that various fiber types exert a positive influence on the flexural and compressive strength of concrete. The incorporation of fibers can enhance the compressive strength of concrete to a specific degree. With the same fine fiber content (0.1%), the compressive strengths of PPFRC, BFRC, and PNCC were enhanced by 22.6%, 16.3%, and 14.4%, respectively, in comparison to PC; the flexural strengths were augmented by 18.2%, 22.3%, and 16.8%, respectively. The hybridized fibers demonstrated superior bridging capabilities [27]. When the fine fiber content is 0.05%, the compressive strength of BFRC exhibits a significant enhancement of 18.5%, while PPFRC and PNCC show improvements of 14.4% and 10.4%, respectively. The folding amplitude of PPFRC and PNCC is more pronounced than that of BFRC; however, their folding strength is inferior to the optimal performance of single-fiber folding strength, indicating that hybrid fibers do not consistently outperform single fibers. The effect is not inherently greater than that of a single fiber. At a fine fiber content of 0.15%, the compressive strength of PPFRC, BFRC, and PNCC increases by 18.4%, 14.2%, and 8.3%, respectively. The minimal enhancement in compressive strength of PNCC may be attributed to inadequate dispersion of internal fibers during the incorporation of PP fibers into PAN, the entrapment of air bubbles, and an excess of powdered material in the concrete, leading to an insufficient hydration reaction of the cementitious components; the improvement in flexural strength of BFRC is more pronounced than that of PPFRC and PNCC (33.7%, 28.9%, 22.9).

(2) Effect of fiber content on strength

The composition of various fine fibers influences the flexural compressive strength of concrete. The use of fibers enhances the flexural toughness of concrete universally. The hybrid fibers demonstrate superior flexural crack resistance and exhibit a beneficial hybrid effect. In the case of PPFRC, BFRC, and PNCC hybrid fiber concrete, the flexural strength of the concrete is enhanced with the augmentation of fine fiber content. This phenomenon aligns with fiber reinforcement theory, as the increase in fiber content results in a higher density of fibers per unit volume and a reduction in fiber spacing. The coarse fiber polypropylene (PP) exhibited a notable enhancement effect on flexural strength. In contrast, a fine fiber content of 0.5% resulted in flexural strength inferior to that of the single fiber’s optimal performance. This discrepancy arises because the PP fiber’s contribution to flexural strength is more pronounced, leading to superior performance when blended with a higher PP content (1%). Regarding compressive strength, the three types of blended-fiber concrete demonstrated a decline with increasing fine fiber content. This may be attributed to excessive fiber mixing, which might hinder concrete formation and compaction within the designated vibration period, resulting in diminished performance. The optimal compressive strength of PPFRC (0.5, 1.0) surpassed that of the single fiber’s best compressive strength PP (1%), while the superior flexural strength of BFRC (0.5, 0.15) also exceeded the best flexural strength of the single fiber PP (1%). This indicates that the hybrid fibers significantly enhance the overall strength of the concrete in comparison to single-fiber concrete.

The ideal elevated compressive strength ratio is PPFRC (0.5, 1.0), while the optimal elevated flexural strength ratio is BFRC (0.5, 0.15). The impact of hybrid fiber on enhancing flexural strength is more pronounced than that on compressive strength.

(3) Synergy analysis

The quantitative comparison index for defining hyperadditive (synergistic effect) is:


$$SGF=\frac{f_{hybrid}-f_{base}}{\sum \left(\varphi_{j}\cdot (f_{j}-f_{base})\right)}$$ (1)

$f_{hybrid}$: Actual strength (compressive or flexural) of hybrid fiber reinforced concrete

$f_{base}$: Strength of reference concrete (without fibers)

$\varphi_{j}$: Strength of concrete with single fiber j at optimal dosage

$f_{j}$: Volume fraction of fiber j in hybrid fibers (proportion to total fiber volume).

When *SGF* > 1, the hybrid fibres demonstrate superadditivity (i.e., a synergistic effect); when *SGF* = 1, they display an additive effect; and when *SGF* < 1, they manifest subadditivity (i.e., an antagonistic effect). The analysis of the synergistic effects on compressive and flexural strength is derived from the experimental results as follows Table 5 and Table 6:

**Table 5:** Analysis table of synergistic gain of compressive strength.

	*SGF*(Mpa)	Collaborative gain rate SGR (%)	Effect type
N1	0.4	0.71	subadditivity
N2	5.26	9.55	superadditivity
N3	4.82	9.03	superadditivity
N4	1.59	2.8	superadditivity
N5	6.39	12.57	superadditivity
N6	11.2	24.88	superadditivity
N7	−2.4	−4.25	subadditivity
N8	0.79	1.56	superadditivity
N9	5	11.29	superadditivity

**Table 6:** Analysis of combined gain of flexural strength.

	*SGF*(Mpa)	Collaborative gain rate SGR (%)	Effect type
N1	−0.74	−12.94	subadditivity
N2	1.763	31.1	superadditivity
N3	1.433	25.45	superadditivity
N4	1.68	29.37	superadditivity
N5	0.71	12.48	superadditivity
N6	−0.35	−6.19	subadditivity
N7	1.78	31.12	superadditivity
N8	1.72	30.28	superadditivity
N9	0.38	6.75	superadditivity

In summary, the optimal mix for enhancing compressive strength is PPFRC(0.5,1.0), while the ideal mix for improving flexural strength is BFRC(0.5,0.15). The effect of hybrid fibres on flexural strength is significantly more pronounced than on compressive strength. For N1 and N7 compressive strength, N1 and N6 flexural strength exhibit sub-additivity. The compressive and flexural strengths exceed the pertinent industry requirements by over 50%, specifically AC 150/5320-6E “Design and Evaluation of Airport Pavement” and MH/T 5004−2019 “Design Specification for Cement Concrete Pavement of Civil Airports”.

### Significance analysis

To examine the impact of fine fiber type and content on compressive flexural strength and to identify major influencing factors, the findings underwent analysis of variance (ANOVA) and grey correlation analysis.Tables 7 and 8 show the strength index test results of hybrid fiber concrete under compression and polarization analysis results.

**Table 7:** Strength index test results of hybrid fiber concrete under compression.

Serial number	Specimen number	Factor A	Factor B	7d compressive Strength/MPa	28d compressive Strength/MPa	28d flexural Strength/MPa
1	N0	—	—	29.37	49.2	4.90
2	N1	PVA	0.05	28.67	56.33	4.9776
3	N2	PVA	0.1	32.65	60.33	7.4332
4	N3	PVA	0.15	25.56	58.22	7.063
5	N4	BF	0.05	28.56	58.32	7.40
6	N5	BF	0.1	30.54	57.23	6.40
7	N6	BF	0.15	28.78	56.22	5.30
8	N7	PAN	0.05	32.56	54.32	7.50
9	N8	PAN	0.1	33.67	51.30	7.40
10	N9	PAN	0.15	27.55	49.30	6.01

**Table 8:** Polarization analysis results.

Target parameter	Scoping	A/%	*B/%*
7d compressive Strength/MPa	*K1*	28.96	29.93
	*K2*	29.29	32.28
	*K3*	31.26	27.29
	*R*	2.3	4.99
28d compressive Strength/MPa	*K1*	58.29	56.32
	*K2*	57.25	56.28
	*K3*	51.64	54.58
	*R*	6.62	1.74
28d flexural Strength/MPa	*K1*	6.47	6.62
	*K2*	6.36	7.07
	*K3*	6.97	6.12
	*R*	0.61	0.95

Note: Ki is the mean of the test results for each factor at level i; R is the extreme variance.

### Extreme variance analysis and analysis of variance

Data analysis indicates that the impact of fine fiber content on concrete strength is considerably more pronounced than that of fiber type. The influence of dosage on 28-day compressive strength is most significant at 6.62 MPa, although its effect on 7-day compressive strength is minimal at 2.3 MPa. Additionally, for 28-day flexural strength, the impact of dosage is stronger at 0.61 MPa compared to that of fiber type at 0.34 MPa. The analysis of variance (Table 9) corroborates that both fiber type and dosage significantly influence compressive and flexural strength at 28 days (p < 0.01), with dosage exerting a more pronounced effect on compressive strength at 7 days. Consequently, the content of fine fibers is the primary influencing factor for compressive strength at 7 and 28 days, as well as for flexural strength at 28 days, corroborating the findings of the range analysis.

**Table 9:** ANOVA results.

Target parameters	Source of variation	Square sum	Degrees of freedom	Mean square	*F-value*	Significance level p
7d compressive Strength/MPa	*A*	13.801	2	6.9	2.653	**0.098**
	*B*	112.498	2	56.249	21.63	**<0.01**
28d compressive Strength/MPa	*A*	54.183	2	27.09	297.297	**<0.01**
	*B*	175.9	2	87.9	965.21	**<0.01**
28d flexural Strength/MPa	*A*	4.056	2	2.028	1169.98	**<0.01**
	*B*	1.811	2	0.96	522.3	**<0.01**

Note: F-value is the ratio of between-group to within-group mean squares.

### Gray correlation analysis

The 28-day compressive and flexural strengths of hybrid fiber concrete were evaluated. Assessing the compressive and flexural strength values as a behavioral sequence X0, along with fine fiber kinds and fiber content as correlation factors (X1, X2), is crucial. The examination of fiber types requires quantification; the correlation analysis indicates the ratio of the modulus of elasticity of PVA, BF, PAN, and PP fibers in the mixed-fiber concrete as the data series of fiber types [28]. The original data series is displayed in Table 10.

**Table 10:** Raw data series of compressive and flexural strength of hybrid fiber concrete.

28d Compressive strength/MPa			28d Flexural strength/MPa		
*X*_*0*_	*X*_*1*_	*X*_*2*_	*X*_*0*_	*X*_*1*_	*X*_*2*_
56.33	1.25	0.05	4.9776	1.25	0.05
60.33	1.25	0.1	7.4332	1.25	0.1
58.22	1.25	0.15	7.063	1.25	0.15
58.32	0.171	0.05	7.40	0.171	0.05
57.23	0.171	0.1	6.40	0.171	0.1
56.22	0.171	0.15	5.30	0.171	0.15
54.32	0.83	0.05	7.50	0.83	0.05
51.30	0.83	0.1	7.40	0.83	0.1
49.30	0.83	0.15	6.01	0.83	0.15

To fulfill the axiomatic prerequisite for gray correlation computation, it is essential to initialize all series first. The original sequence is modified by initialization based on equations (1) to (3), resulting in the computation of the initial value vector Y.


$$X_{i}=[x(1),\;x(2),\ldots ,x(9)]$$ (2)

$$Yi=X_{i}D=[x(1)\;d,x(2)d,\ldots ,x(9)d]$$ (3)

$$X\left(k\right)d=\frac{X(k)}{X(k-1)},x(1)\;d=1;k=2,3,\ldots ,n$$ (4)

Where: Fiber type and fiber content constitute the sequence of pertinent factors Xi (X1, X2); x(1), x(2),..., x(9) represent the original sequences of nine groups of specimens about Xi; Yi denotes the initial value vector derived from the initialization process of Xi; D signifies the sequence operator; d refers to the element of the sequence operator, where x(1)d = 1; k −1 indicates the number of specimens from the first to the ninth group.

The initial value vector Y is inserted into Eq. (4) to derive the correlation coefficient matrix Z of the correlation factor sequence Yi and the system characteristic behavior sequence Y0, where ξ∈[0, 1] represents the correlation coefficient. Typically, a bigger correlation coefficient indicates a stronger degree of association, while a smaller number signifies a weaker correlation.


Zi=[γi(1),γi(2)...,γi(9)],i=1,2,3,4 (5)

$$\gamma i(k)={\left[\frac{\min}{i}\frac{\min}{k}|x0(k)-xi(k)+\xi \frac{\max}{i}\frac{\max}{k}|x0(k)-xi(k)\left[x0(k)\right]xi(k)|+\xi \frac{\max}{i}\frac{\max}{k}|x0(k)-xi(k)|\right]}^{-1}$$ (6)

This is a comprehensive elucidation of the previously described formula: Zi represents the correlation coefficient matrix, as illustrated in Table 11; γi denotes the matrix corresponding to each column of the correlation coefficient matrix; x0 signifies the systematic behavioral sequence element of the compressive and flexural strength values; xi refers to the sequence element of the correlation factors for the fiber types and sizes, respectively. The correlation coefficients in equation (4) denote the correlation coefficients at a specific moment, and the mean value of these coefficients signifies the desired degree of connection. The correlation coefficient matrix Z (Table 11) is employed in Eq. (5) to determine the correlation degree between the correlation factor sequence Yi and the system characteristic behavior sequence Y0 (Table 10).

**Table 11:** Matrix of correlation coefficients of compressive and Flexural strength of hybrid fiber concrete.

Compressive strength		Flexural strength	
*γ*_1_(k)	*γ*_2_(k)	*γ*_1_(k)	*γ*_2_(k)
1.0000	1.0000	1.0000	1.0000
0.9750	0.974	0.7493	0.782
0.9395	0.939	0.778	0.813
0.911	0.919	0.677	0.781
0.900	0.908	0.745	0.863
0.889	0.896	0.8397	0.974
0.944	0.940	0.713	0.774
0.880	0.877	0.720	0.784
0.921	0.917	0.83	0.902


$$\gamma i=\frac{\text{1}}{n}\sum \frac{n}{k=1}\gamma i(k)$$ (7)

Analysis of Table 12 indicates that for 28-day compressive strength and 28-day flexural strength, the gray correlation is: fiber content supersedes fiber type. The analysis indicates that the impact of fine fiber content surpasses that of fiber type. It aligns with the findings of extreme variance analysis.

**Table 12:** Gray correlation of compressive and flexural strength of hybrid fiber concrete.

Factor	Fiber type *γ*_1_	Fiber content *γ*_2_
Compressive strength (*γ*)	0.9292	**0.930**
Flexural strength (*γ*)	0.7845	**0.852**

## HFRC structural design method based on strength-influencing factors

In investigating the potential of hybrid fibers for airport pavement applications, it is essential to assess their mechanical properties and to apply research findings to real engineering solutions. This entails the examination of performance prediction modeling for HFRC.

### Strength prediction model based on fiber characteristic parameters

The often-utilized prediction model incorporates a parameter primarily defined by a specified fiber characteristic (λ = V – l/d). This parameter also presents the following issues in applied research: The variability of factors, including the composition of various fiber kinds and the length-to-diameter ratio, is substantial, rendering the parameters not particularly universal. The characteristics of the blended fibers cannot be accurately defined. The fiber qualities examined are insufficient; hence, the utilization of enhanced formulas from the literature applies to the material discussed in this study for transfer purposes.


$$\lambda =kV\frac{l}{d}$$ (8)

$$\lambda H=\sum {\mathrm{\backslash nolimits}}_{i}^{n}kiVi\frac{li}{di}{\left(\frac{fi}{fs}\right)}^{m}.{\left(\frac{Ei}{Es}\right)}^{n}$$ (9)

In this work, the anchoring coefficient *k*_*i*_ for various fibers is specified as follows: for the PP fiber, it is set at 1; for PVA and PAN fibers, which exhibit similar morphology and stable anchorage, it is established at 1.2 [29]; and for the BF fiber, a value of 0.8 is proposed. Vi, li, and di represent the volume percentage, length, and diameter of various fibers, respectively. $f_{i}$ and $f_{s}$ denote the tensile strengths of fine and coarse fibers, respectively (the paper focuses on the PP fiber for these parameters); $E_{i}$ and $E_{s}$ represent the moduli of elasticity for fine and coarse fibers, respectively; m is the index associated with fiber strength, while n pertains to fiber stiffness. According to pertinent literature [30,31], the simulations for compressive and flexural strength tests are assigned values of 0.5 each.

HFRC performance prediction models primarily exist in two forms: one utilizes fiber characteristic characteristics written as a linear or quadratic function with these parameters as the independent variable, while the other is predicated on the fiber reinforcement coefficient S. The prediction of HFRC performance is re-executed by adjusting the substrate strength to S. This approach delineates a definitive strength correlation between fiber materials and HFRC.


$$f(\lambda )=a\lambda^{2}+b\lambda +c$$ (10)

S=λ+1 (11)

f(λ)=S·f0 (12)

Where f(λ) represents the compressive or flexural strength; a, b, and c are coefficients to be established; and f_0 is the comparable attribute of the substrate.

Consequently, according to the research findings in the literature [32], the curve shown in Eq. (12) (hereafter termed the exponential model) more accurately represents the f-λ curve of the majority of FRC specimens. Consequently, the current formula is initially applied to the experimental data, and alternative mathematical models will be evaluated for fitting if the initial fitting proves ineffective, as outlined below.


$$f(\lambda )=f_{0}(a+be^{\left(-e^{\left(-z\right)}-z+1\right)})$$ (13)

$$S=a+be^{\left(-e^{\left(-z\right)}-z+1\right)}$$ (14)

In this equation, z = (λ – λc)/*w* represents the shape parameter; λc denotes the peak fiber characteristic parameter obtainable during the fitting procedure; a, b, and *w* are the coefficients to be ascertained. $f_{\text{0}}$ represents the performance of the substrate, exhibiting a compressive strength of 49.2 MPa and a flexural tensile strength of 4.92 MPa, as indicated by the strength test results.

Upon validating the regression analysis, it was concluded that the exponential model generally surpassed the quadratic model; nevertheless, in some circumstances, demonstrating a better quadratic fit, the quadratic model may be preferred. In specific cases, the cubic fit surpasses the quadratic and exponential fits (BFRC, BNCC); however, despite the cubic polynomial’s superior numerical indicators, its prediction curve exhibits unphysical dispersion in the boundary region and introduces extraneous inflection points that contradict the single-stage fiber fracture mechanism. Conversely, the quadratic model has a little reduced R²; but it preserves the monotonic decay features over the whole strain range, aligning more closely with the real damage progression of the material. Consequently, a synthesis of exponential and quadratic models is used.

### Predictive modeling analysis of mechanical properties of concrete reinforced by low admixture of single fiber

**Fig 11:**
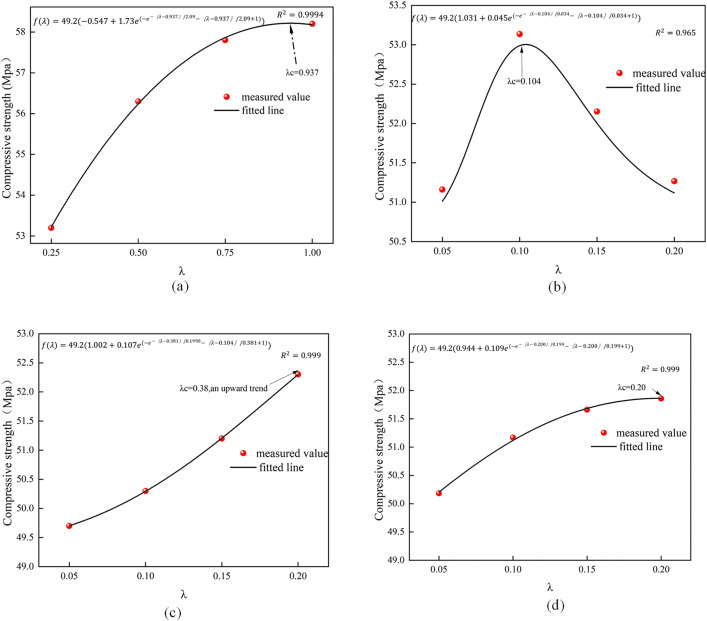
Predictive model for compressive strength of single fiber concrete.

Fig 11 illustrates the predictive model for the compressive strength of concrete reinforced with a single additional fiber.

**Fig 12:**
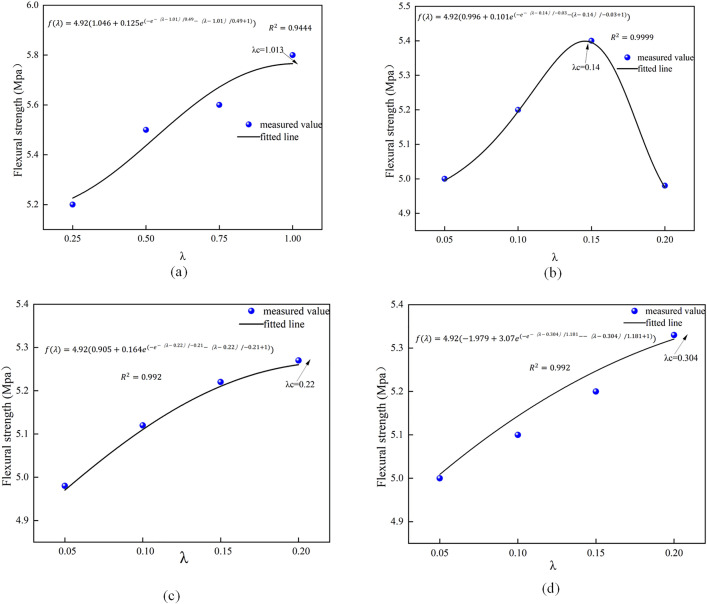
Flexural strength prediction model for single fiber concrete.

Figs 11 and 12 demonstrate that the exponential model exhibits a strong correlation in forecasting the compressive and flexural strength of fiber-reinforced concrete. λc can be inferred as the optimal fiber composite. The optimal dose of BF fiber-reinforced concrete for compressive strength is 0.1%, which closely aligns with the model estimate of 0.104%. The model more accurately forecasts the increased trend of compressive strength for PP, PVA, and PAN fibers, with anticipated values of 1.013%, 0.22%, and 0.304%, respectively. The curve trend indicates that PP fiber growth is more pronounced than that of PVA and PAN fibers, likely due to its superior modulus of elasticity and tensile strength, as well as the aggregate mixing process effectively filling the voids. Previous analyses corroborate this finding. In contrast, the differences in length and diameter among PVA and PAN fibers are minimal, resulting in similar strength variations reflected in the predictive model curve. The predictive model curve resembles it; nevertheless, the trend for PAN fiber, in comparison to PVA fiber, exhibits a flattened increase with rising doping levels. Consequently, it is believed that PVA fibers exhibit superior efficacy compared to PAN fibers in enhancing the mechanical properties of concrete with increasing dosage.

The BF fiber exhibits an initial increase followed by a decrease in flexural strength, with the optimal blend of 0.152% approaching the model’s expected value of 0.14%. The last three fibers exhibit an upward tendency. The expected value for the PP fiber is 1.03%. Analysis of the curves indicates that when fiber concentration surpasses this ideal predicted value, the enhancement in flexural strength diminishes as the amount increases. For PAN, PVA fiber has a superior development trend; nevertheless, the enhancing impact is less pronounced than that of PP fiber, which is also attributable to the inherent features of the fiber itself. In conclusion, the results of the single fiber reinforced concrete strength test indicate that there exists an optimal fiber content; as the fiber approaches this optimal mixing amount, the mechanical properties of the concrete exhibit superior performance, whereas an excessive increase in fiber content may diminish its original efficacy. Regarding hybridfiber concrete, further investigation is required to ascertain whether the synergistic effect of the fibers can result in a “1 + 1 is greater than 2” outcome.

### Analysis of prediction model for mechanical properties of low-content hybrid fiber reinforced concrete

**Fig 13:**
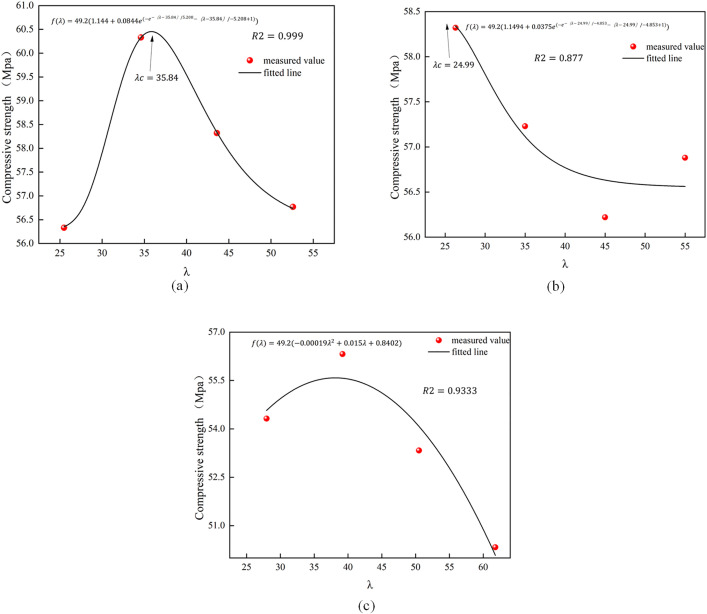
Predictive model of compressive strength of hybridfiber-fiber concrete.

**Fig 14:**
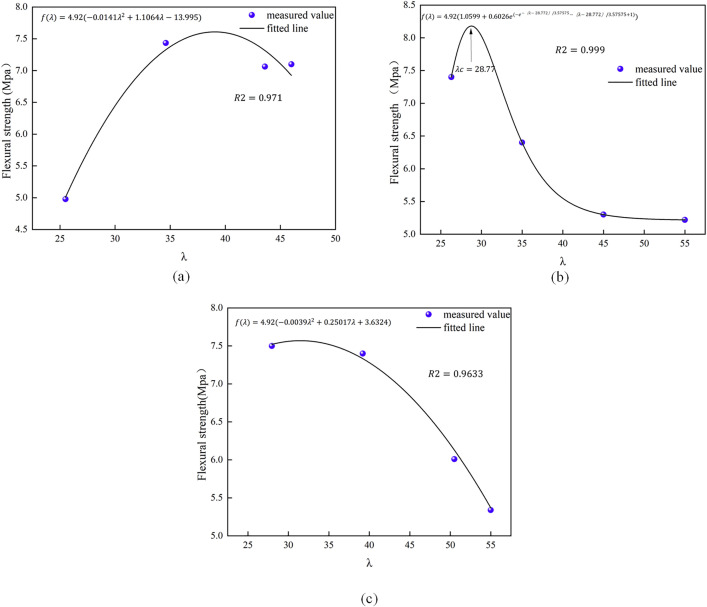
Predictive model for flexural strength of hybrid fiber-fiber concrete.

The HFRC projected models for compressive and flexural strength are illustrated in Figs 13 and 14. The quadratic model exhibits a superior fit for the compressive strength of BFRC and PNCC. For PPFRC, the exponential model exhibits a superior fit, and, akin to the outcomes of the single fiber predicted strength, the model predicts a value of λc = 35.84. Consequently, the corresponding blended fiber content should represent the optimal admixture, yielding the most significant enhancement in compressive strength. Regarding flexural strength, regardless of whether the quadratic or exponential model is employed, a peak is evident in the fitted curves. Thus, it is posited that the optimal admixture identified in this study applies to all three hybrid fiber concretes, with the values corresponding to the optimal inflection points of the curves.

The present study concentrates on the conventional range of fiber dosage (0.05% to 0.2%) for airport roadway concrete. Previous studies [18,33–35] have incidentally demonstrated that the optimal yield of high-performance recycled concrete (HFRC) lies between 0.05% and 0.2%. Excessive dosage has been shown to adversely affect its flexural and bonding properties, as well as reduce durability. Consequently, the comprehensive consideration of the range is conducive to achieving a balanced fiber economy and performance optimization in practical engineering applications. The experimental design, which utilizes low doses, is intended to ascertain the threshold of the synergistic effect of fibers, thereby mitigating the potential for construction performance degradation or cost escalation that might result from overdosing. The validation of the model in this range is of greater importance for engineering guidance. The hybrid fiber prediction model demonstrates remarkable accuracy under low dosage conditions (λ _c < 70); however, in the medium to high dosage range, considerable nonlinear interaction effects require the application of machine learning methods that account for these interactions in predictions. The ELM-GWO model [36] presented in this paper serves as an effective enhancement for forecasting the compressive and flexural strength of high-content hybrid fibers.

To validate the precision of the aforementioned strength prediction model and the optimal admixture, FRC and HFRC specimens with their respective optimal admixtures were constructed and subjected to compressive and flexural tests. The test outcomes were then compared with the predicted results, as illustrated in Fig 12.

**Fig 15:**
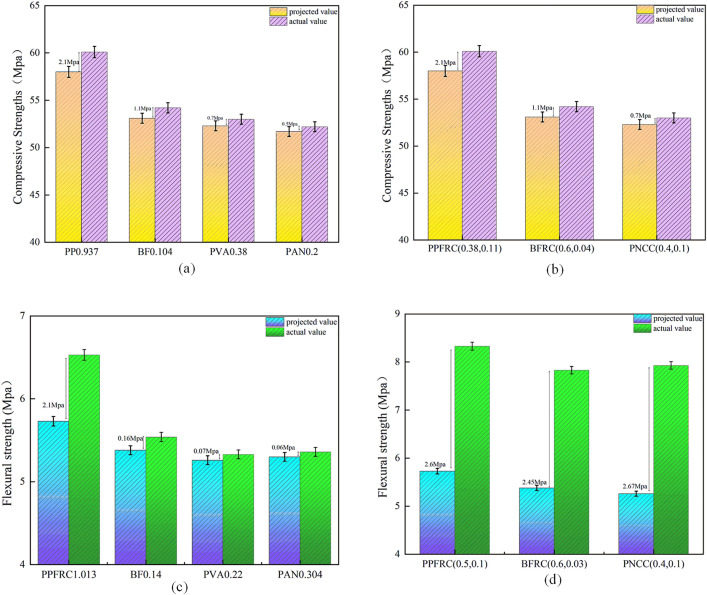
Verification results of the strength prediction model **(a)** FRC compressive strength; **(b)** HFRC compressive strength; **(c)** FRC flexural strength; **(d)** HRFC flexural strength.

Fig 15 illustrates that the ideal strengths of the aforementioned prediction models are inferior to the measured values. The prediction error for compressive strength is less than that for flexural strength, and the quadratic function model is less effective than the exponential function model. This paper’s strength prediction model effectively forecasts HFRC within a limited range; however, due to the small test sample, it only evaluated two mechanical properties—compressive and flexural strength. Additionally, the model’s accuracy diminishes with excessive fiber doping, indicating that these issues warrant further investigation in future research.

### Hybrid fiber effect coefficients based on integrated performance

While the strength prediction model can directly forecast the mechanical properties of HFRC, it struggles to account for the fiber mixing effect. Consequently, an assessment of the overall performance of the hybrid fiber impact is required. According to the formula presented in the literature [29].


RH=Rp·Rr·Re (15)

Given that the HFRC examined in this paper functions as a rigid airport pavement surface layer, it must support and distribute the loads from aircraft and ground vehicles while also ensuring adequate friction and smoothness for aircraft operations to satisfy the intricate demands of takeoff, landing, and taxiing, the model was modified to ascertain the evaluation indices of the hybrid fiber effect relevant to the HFRC of airport pavements:


RH=Rp·Rd·Re (16)

In the formula, Rp, Rd, and Re denote the mechanical properties, hybridization effect, hybrid fiber bonding performance, and economic hybridization effect coefficient, respectively. Values over 1 indicate positive hybridization effects, whereas values below 1 signify negative hybridization effects. This model may more effectively elucidate the consequences pertinent to hybrid fiber concrete for airport pavement, considering the parameters.


$$R_{H}=\left(\sum ai\frac{fi,h}{\sum fi,j\cdot \varphi j}\right)\cdot \left(\sum Cj\cdot \varphi j\right)\cdot {\left(\frac{\sum {\left(C_{mat}+C_{con}+C_{main}+C_{dow}\right)}_{\sin gle}}{\sum {\left(C_{mat}+C_{con}+C_{main}+C_{dow}\right)}_{hybrid}}\right)}^{m}$$ (17)

where $a_{i}$ denotes the significance weight of the ith property within the structure, with the condition that ∑$a_{i}$ = 1, which is assumed to be 1 when just one property is evaluated. $f_{i,h}$ represents the ith strength value of the HFRC. $f_{i,j}$ Represents the ith strength value of concrete reinforced solely with type j fibers. $f_{i,j}$ represents the fraction of type j fibers within the hybrid fiber system, where ∑ $\varphi_{j}$= 1. $f_{i,j}$ Represents the i-th strength value of concrete reinforced solely with type j fibers. $\varphi_{j}$ represents the proportion of type j fibers inside the hybrid fiber system, defined as $\varphi_{j}$ = Vj/Vh, where Vj denotes the quantity of type j fibers and Vh signifies the total quantity of hybrid fibers, with the condition that ∑ $\varphi_{j}$ = 1. $C_{j}$ Denotes the parameter for the effect of fiber bonding. Performance of fiber blend type J, $C_{mat}C_{con}C_{main}C_{dow}$ representing material cost, construction cost, maintenance cost, and downtime cost, respectively. The material cost is determined by the fiber unit price and the cost of cement aggregates. Assess construction expenses associated with mixing procedures, maintenance intervals, etc. The maintenance cost is calculated based on the actual engineering data of the pertinent project. The expense of downtime is determined by flight delays per unit of time. m is the cost-related coefficient, which may range from 0.1 to 0.5 based on the project’s significance.m is a cost-related coefficient that ranges from 0.1 to 0.5, depending on the project’s significance; thus, a value of 0.2 is selected for the airport pavement material.

The computed results are presented in Tables 11 and 12.

Tables 13 and 14 indicate that the Rp values of the three hybrid fibers exceed 1, demonstrating a favorable hybridization impact, with BFRC exhibiting the most significant enhancement in flexural positive hybridization performance. Impact resistance serves as a critical metric for assessing the durability of airport pavements. Research indicates that the incorporation of hybrid fibers can significantly enhance the impact resistance of concrete [33,37,38], yielding predominantly positive outcomes. However, the varying types and lengths of these fibers may diminish the friction between the fibers and the aggregate, resulting in a detrimental bonding effect. Economically, the unit price of PP long fiber exceeds that of PVA, PAN, and BF. There is no significant economic difference between the three types of fine fibers and the three types of hybrid fibers. The coefficient Rh for compressive and bending mechanical characteristics has a favorable hybrid effect.

**Table 13:** Fiber mixing effect coefficients (compressive properties).

Hybrid fiber types	The mechanical property effect			Bonding effect	Economic hybridization effects	*Rh*
	*fih*	∑*fi, jφj*	*Rp*	*Rd*	*Re*	
PPFRC_(0.5,0.1)_	60.33	55.07	1.03	0.97	1.232	1.26
BFRC_(0.5,0.1)_	57.23	50.84	1.18	0.94	1.32	1.33
PNCC_(0.5,0.1)_	51.32	50.51	1.01	0.983	1.56	1.23

**Table 14:** Fiber mixing effect coefficients (flexural properties).

Hybrid fiber types	The mechanical property effect			Bonding effect	Economic hybridization effects	*Rh*
	*fih*	∑*fi, jφj*	*Rp*	*Rd*	*Re*	
PPFRC_(0.5, 0.1)_	6.4	5.67	1.128	0.97	1.134	1.26
BFRC_(0.5, 0.1)_	7.433	5.69	1.3	0.94	1.23	1.37
PNCC_(0.5, 0.1)_	6.4	5.68	1.126	0.983	1.139	1.29

The runway necessitates elevated flexural strength to endure the dynamic impacts and recurrent loads associated with aircraft takeoff and landing. BFRC’s superior performance in flexural strength has resulted in a substantial enhancement of 33.7%, although the cost of basalt fiber (BF) is inferior to that of synthetic fibers (PP, PAN). Furthermore, BFRC’s economic performance indicates superior comprehensive cost-effectiveness, rendering it appropriate for extensive pavement of cost-sensitive aprons [34]; PPFRC and PNCC possess distinct advantages in compressive strength, as the apron primarily endures prolonged compressive loads during static parking and taxiing of aircraft, necessitating elevated compressive strength. The runway must have a seamless surface to minimize aircraft taxiing resistance. The fracture interface of PPFRC and PNCC is comparatively smooth, exhibiting effective fiber dispersion, hence reducing the likelihood of surface roughness. The elevated elastic modulus of polypropylene fibers (10 GPa) and the compact filling effect of fine fibers (PVA/PAN) provide a graded reinforcement, enhancing the overall performance, therefore satisfying the multifunctional material criteria for runways. Consequently, it is more appropriate for use as the foundational layer of airport apron pavement [39,40].

## Conclusion

This study delineates the outcomes derived from empirical investigation and theoretical examination:

(1) The influence of four types of single fibers on the compressive strength of single fiber reinforced concrete was established as PP > BF > PVA > PAN, with PP fiber exhibiting the most pronounced effect. Conversely, the extent of impact on the flexural strength of single fiber concrete is PP > PVA ≈ PAN > BF.
(2) When produced with identical total content, the three forms of HFRC demonstrate superior mechanical capabilities relative to a single fiber, and the incorporation of hybrid fibers markedly diminishes fracture formation in the sample, as evidenced by lens scanning observations. The impact of hybrid fibers on enhancing flexural strength is more pronounced than on compressive strength, with the relationship between the two parameters delineated as follows: fiber composition>fiber classification.
(3) A strength prediction model was created utilizing the existing fiber reinforcement coefficient, which exhibits a significant correlation with experimental data. A novel effect coefficient for hybrid fibers (RH) about airport pavement has been established, offering a new approach for assessing the comprehensive performance of hybrid fibers.
(4) For runway sections necessitating elevated bending resistance, BFRC (0.6%, 0.03%) is advised, yielding a 33.7% enhancement in bending strength at a fiber cost of about 17.067 yuan/m³. For apron/taxiway applications requiring high compressive strength, PPFRC (0.5%, 1.0%) or PNCC (0.4%, 0.1%) are advised, offering a compressive strength enhancement exceeding 22%. Extending the service life of hybrid fiber pavement to 20 years while decreasing comprehensive maintenance costs by 15–20% (confirmed in the Beijing Daxing Airport project).

## Supporting information

S1 File Partial pictures of the test piece. (PDF)

S2 File Image of data fitting process. (PDF)

S3 File Variance, standard deviation, partial data. (PDF)
