# Supplementary material for: Research on mechanical properties and prediction methods of hybrid fiber concrete for airport pavements
Source: PLoS One. 2025 Nov 11;20(11):e0331951. doi: 10.1371/journal.pone.0331951 (PMC12604807; doi:10.1371/journal.pone.0331951)

S1 Partial pictures of the test piece

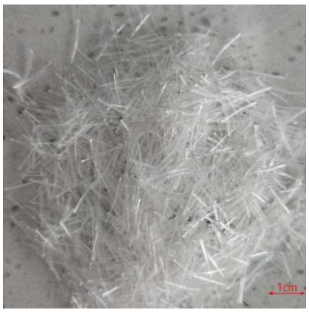

（a） Synthetic coarse polypropylene fibers （PP）

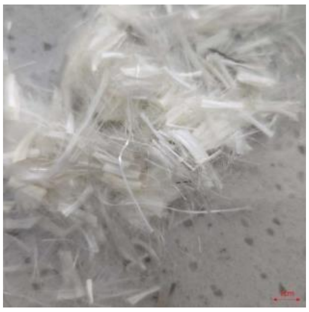

（b） Polyvinyl alcohol (PVA) synthetic fibers

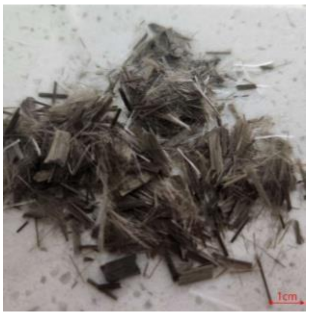

(c) Modified impregnated basalt fiber (BF)

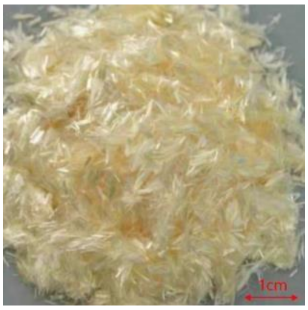

(d) Polyacrylonitrile (PAN) synthetic fibers

Figure 1 Schematic of fiber appearance

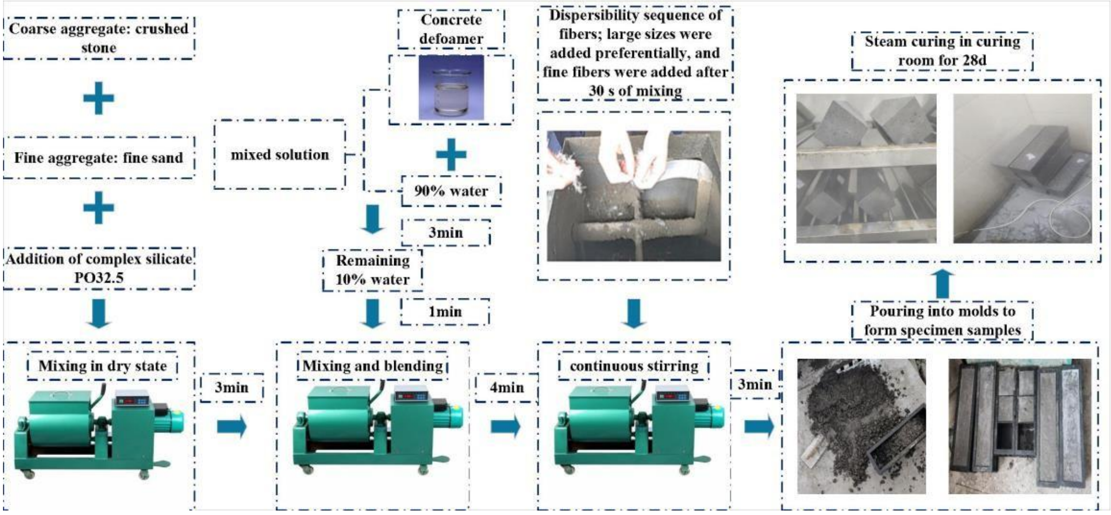

Fig. 2 Flow chart of sample preparation

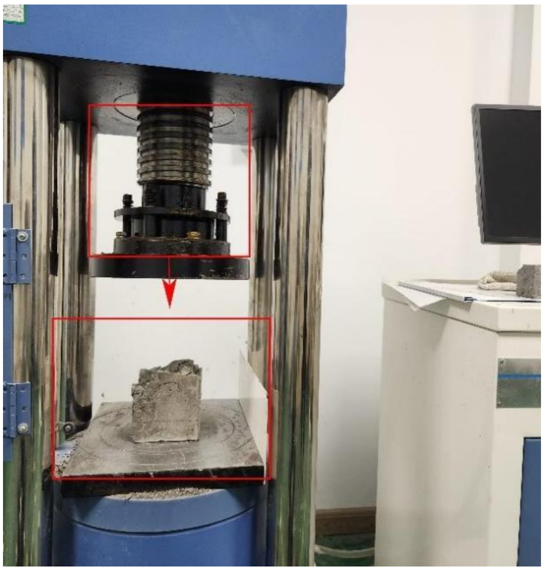

Figure 3 Compressive damage site of the specimen

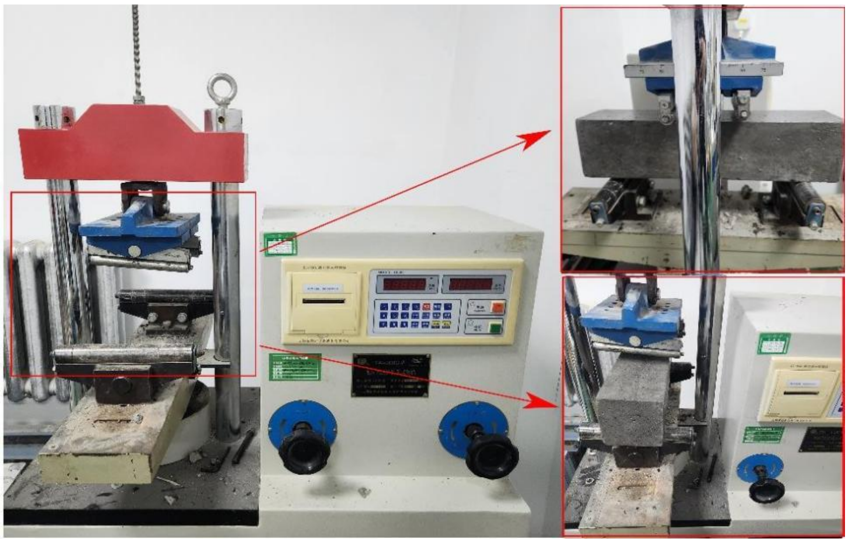

Figure 4 Flexural toughness loading device and field

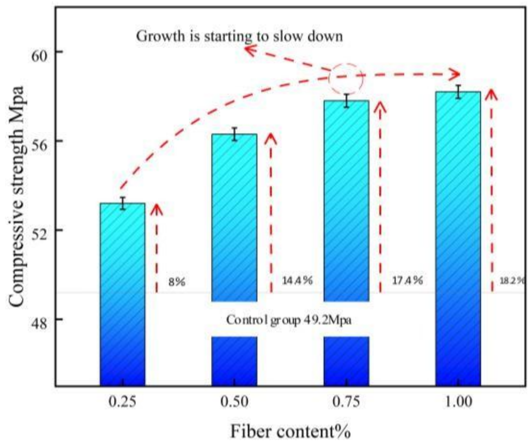

（a）PP

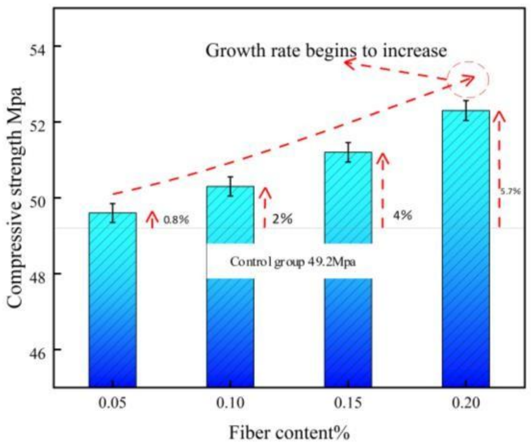

（b）PVA

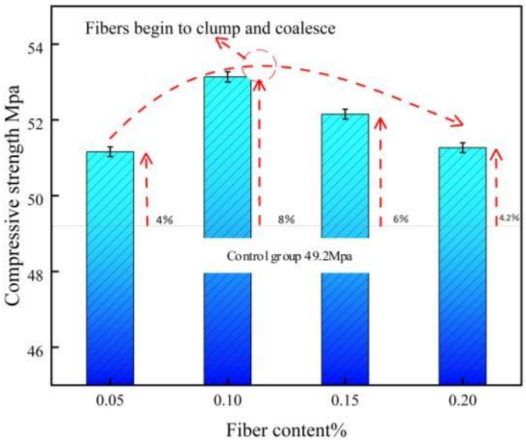

（c）BF

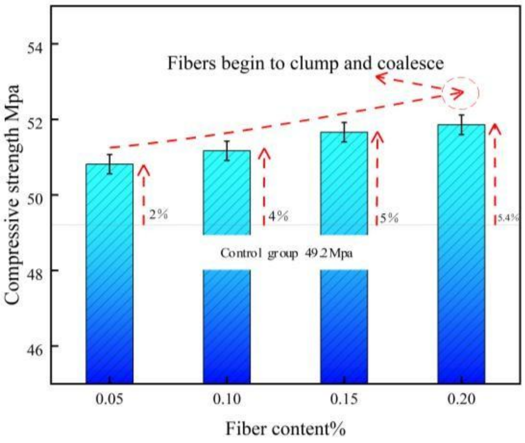

（d）PAN

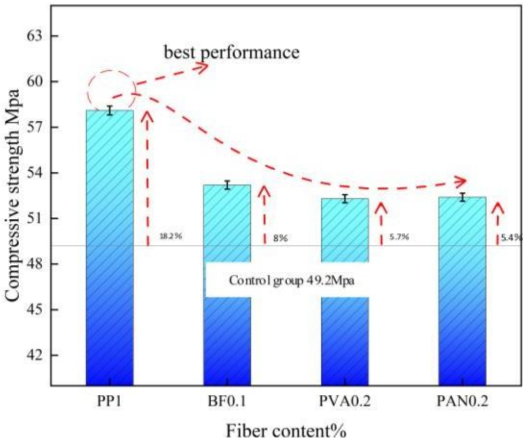

(e) Comparison of optimal values for each group of samples

Figure 5 Role of various types of fibers in increasing the compressive strength of concrete

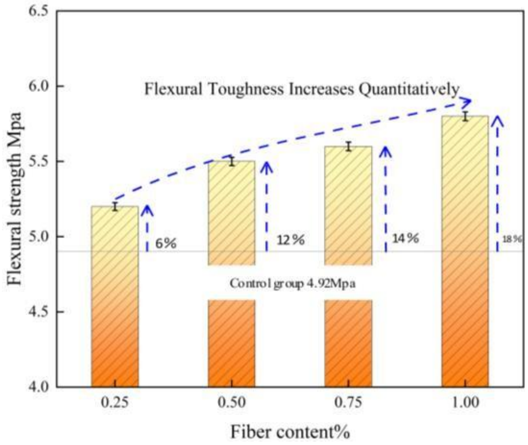

（a）PP

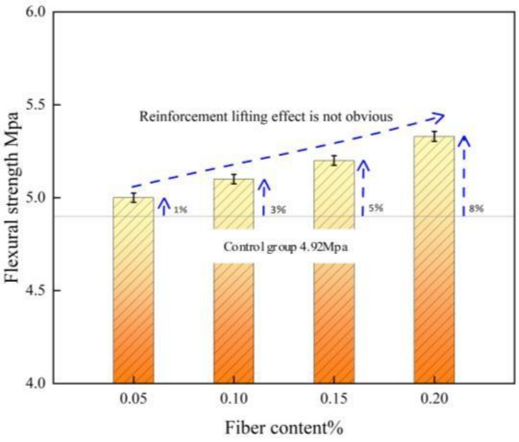

（b）PVA

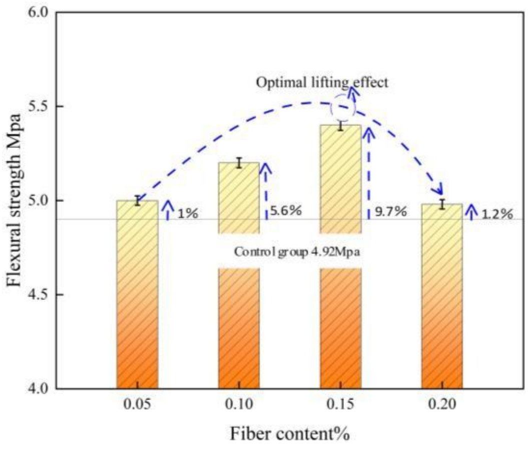

（c）BF

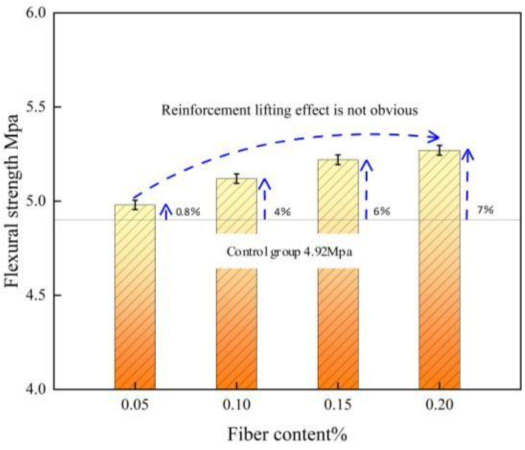

（d）PAN

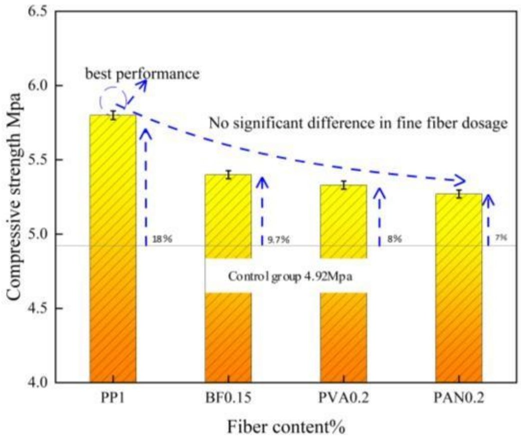

（e）Comparison of optimal values for each group of samples

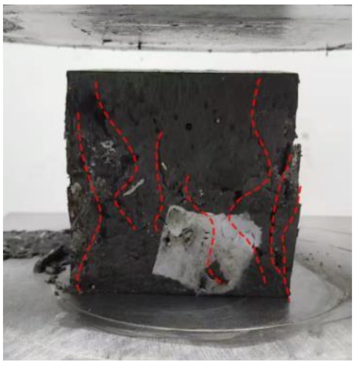

（a）N0

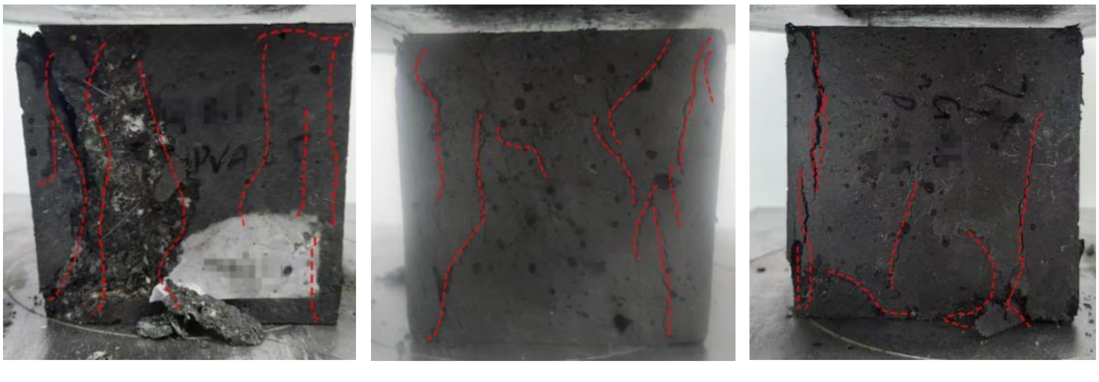

（b）N1 （c）N2 （d）N3

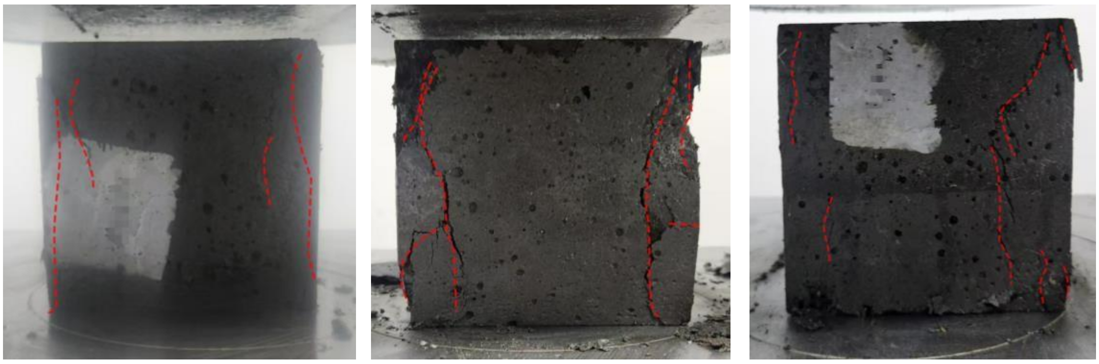

（e）N4 （f）N5 （g）N6

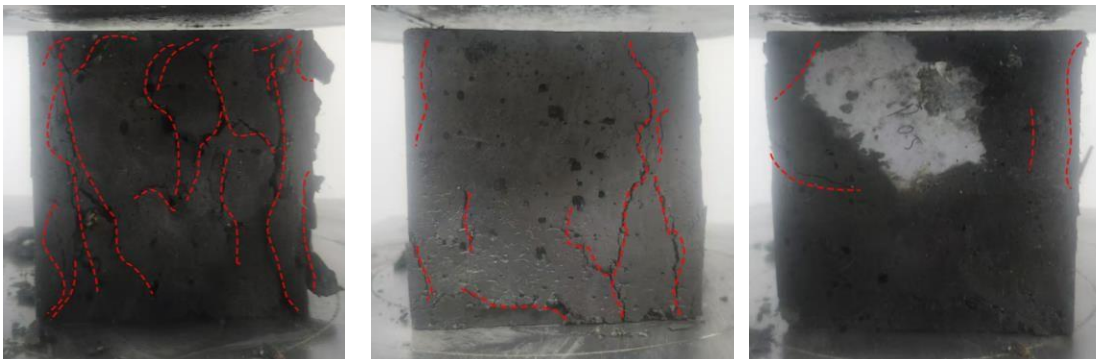

（h）N7 （i）N8 （j）N9

Fig. 7 Compressive damage patterns of mixed fiber concrete and ordinary concrete specimens **(a~j)**

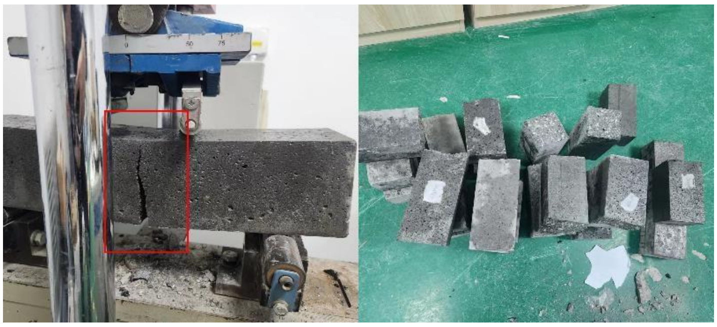

Figure 8 Flexural toughness damage pattern

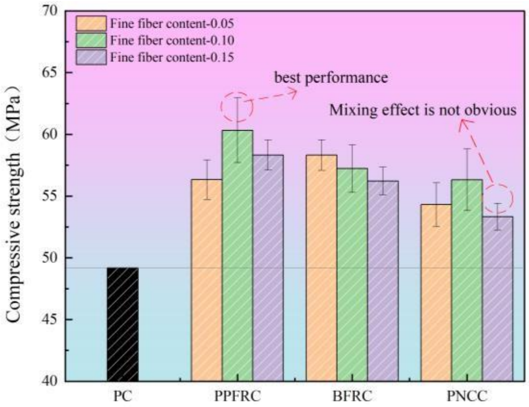

（a）compressive strength

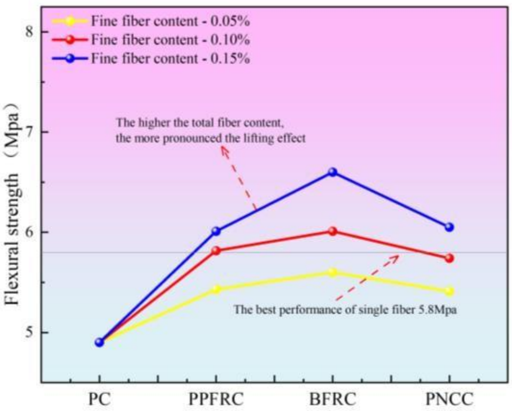

（b）flexural strength

Figure 9 Plot of HFRC mechanical property data

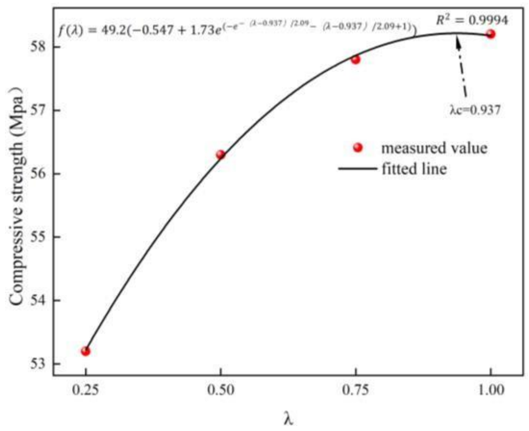

（a）PP

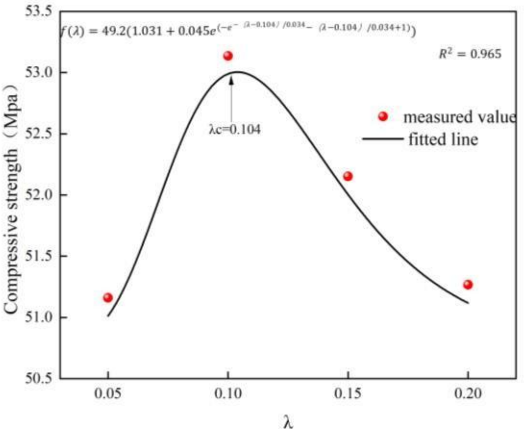

（b）BF

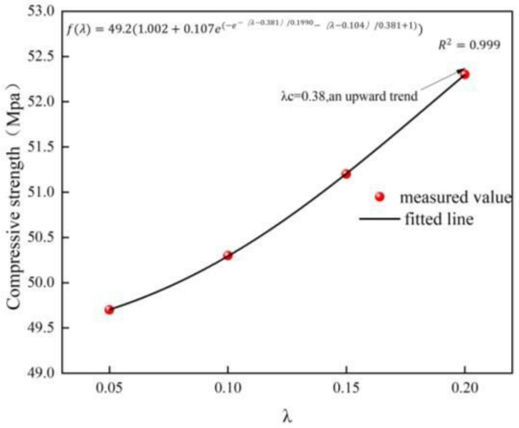

（c）PVA

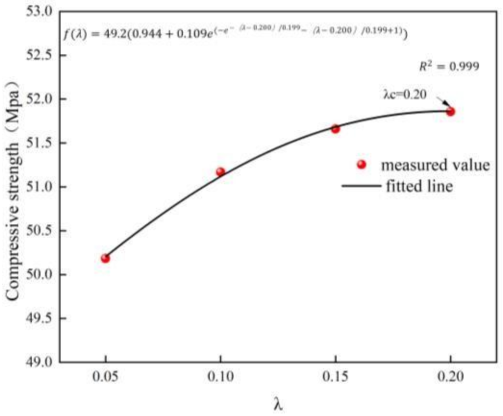

（d）PAN

Figure 10 Predictive model for compressive strength of single fiber concrete

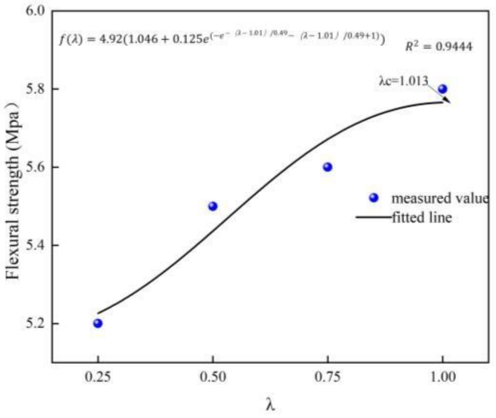

（a）PP

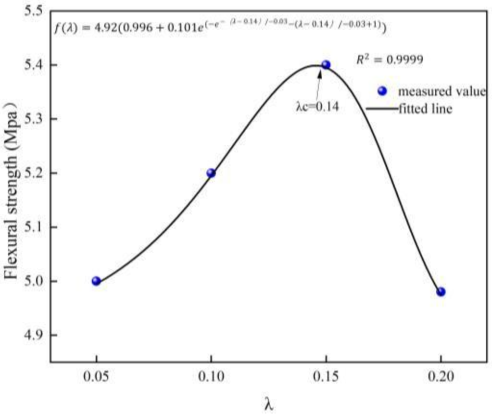

（b）BF

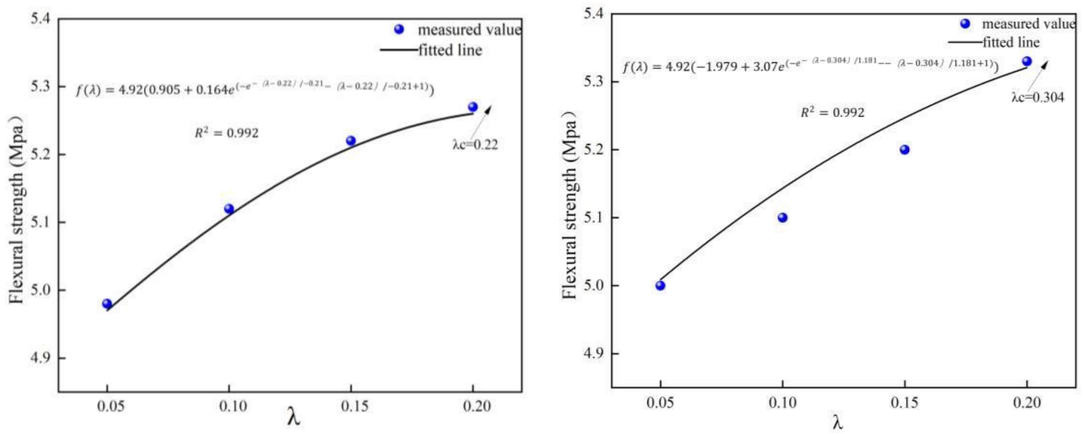

（**c**）PVA （**d**）PAN

Figure 11 Flexural strength prediction model for single fiber concrete

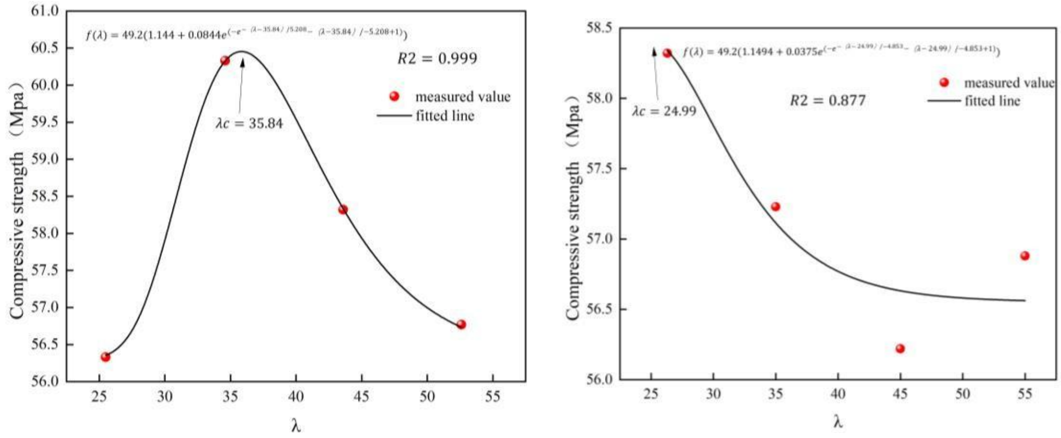

（**a**）PPFRC （**b**）BFRC

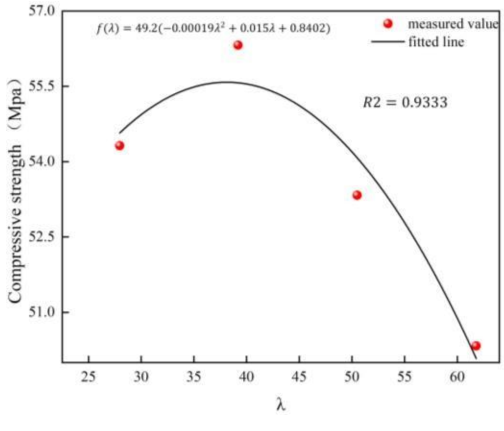

(c) PNCC

Figure 12 Predictive model of compressive strength of mixed fiber-fiber concrete

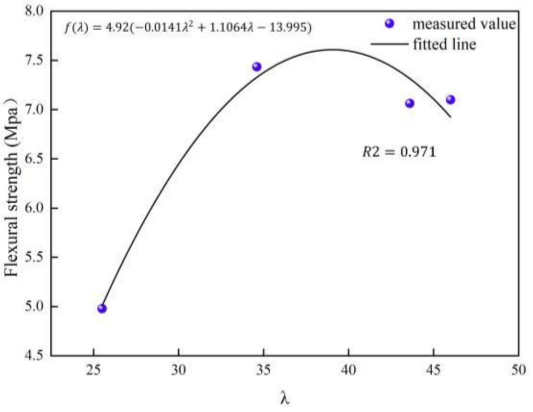

（**a**）PPFRC

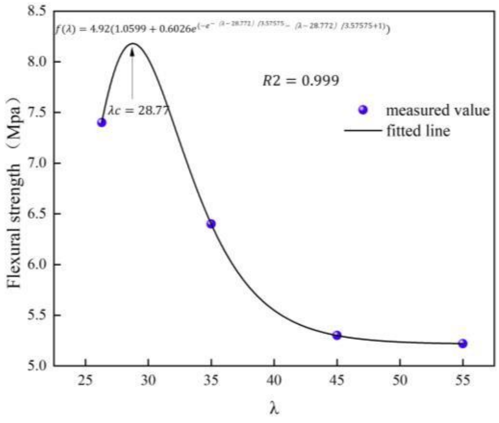

（**b**）BFRC

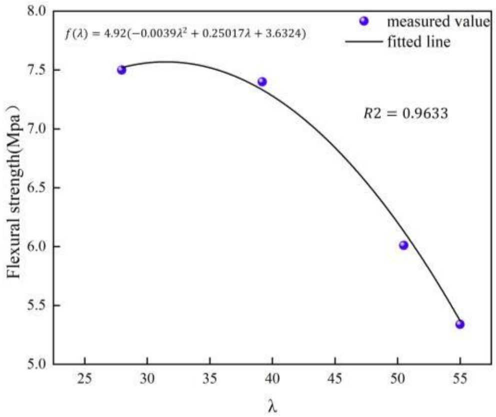

(c) PNCC

Figure 13 Predictive model for flexural strength of mixed fiber-fiber concrete

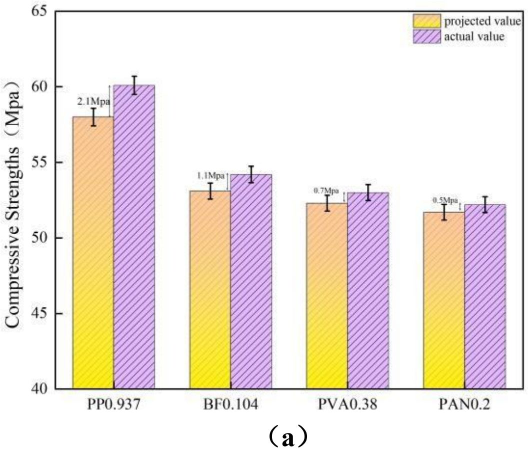

（**a**）

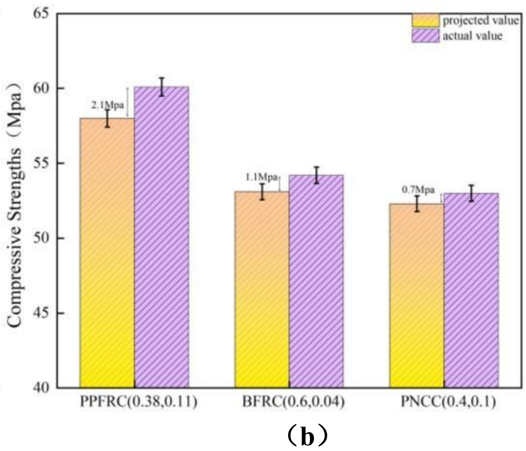

（**b**）

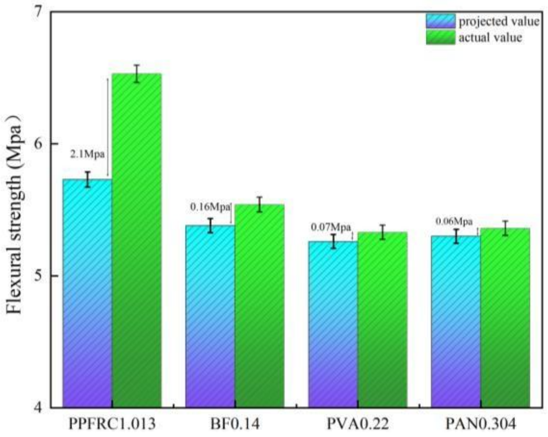

（c）

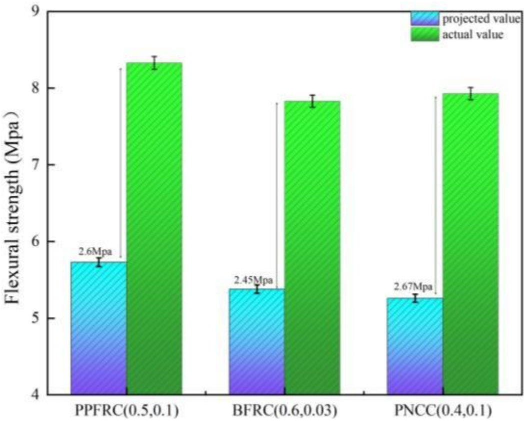

（d）

Figure 14 Strength prediction model certification

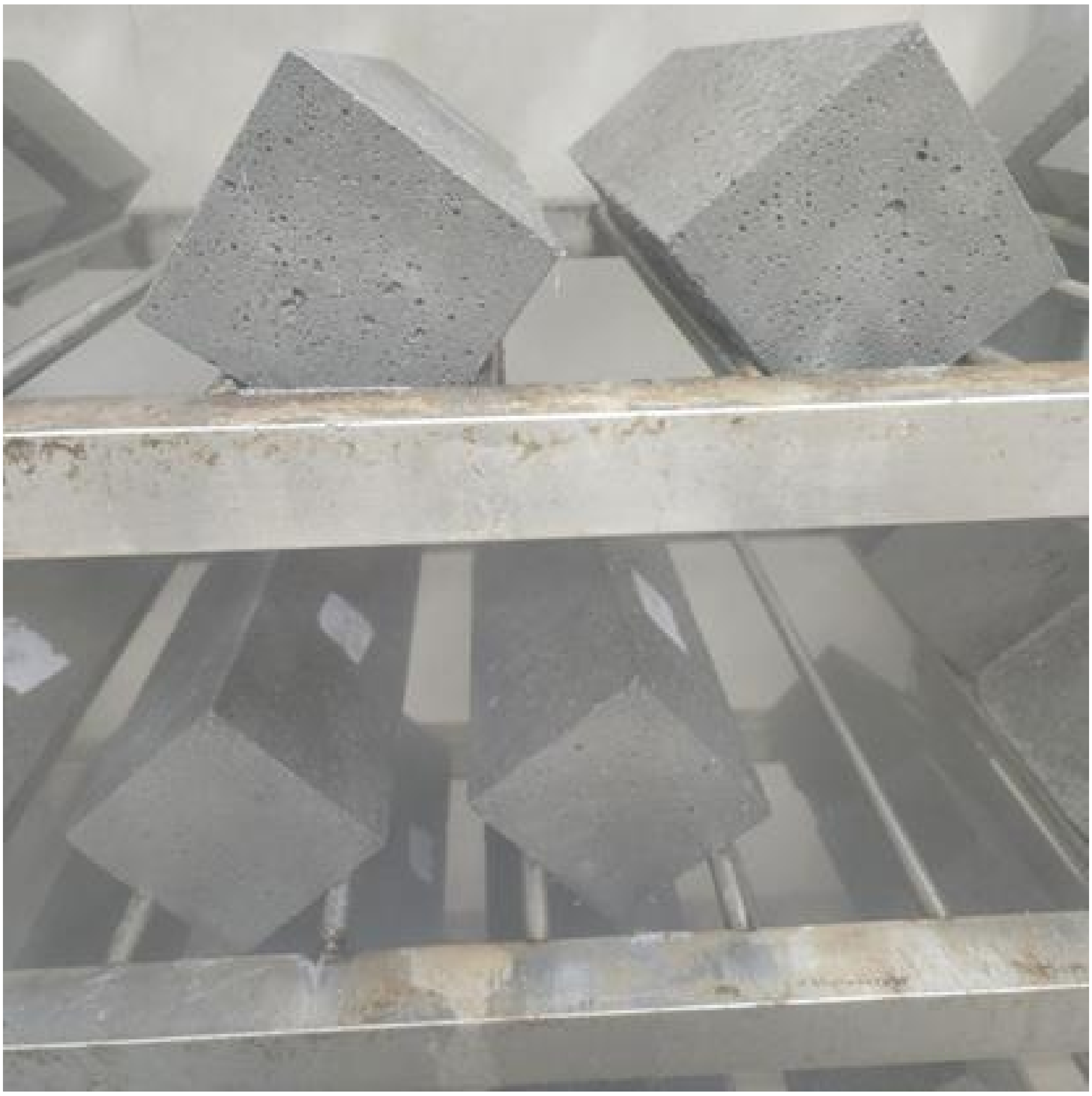

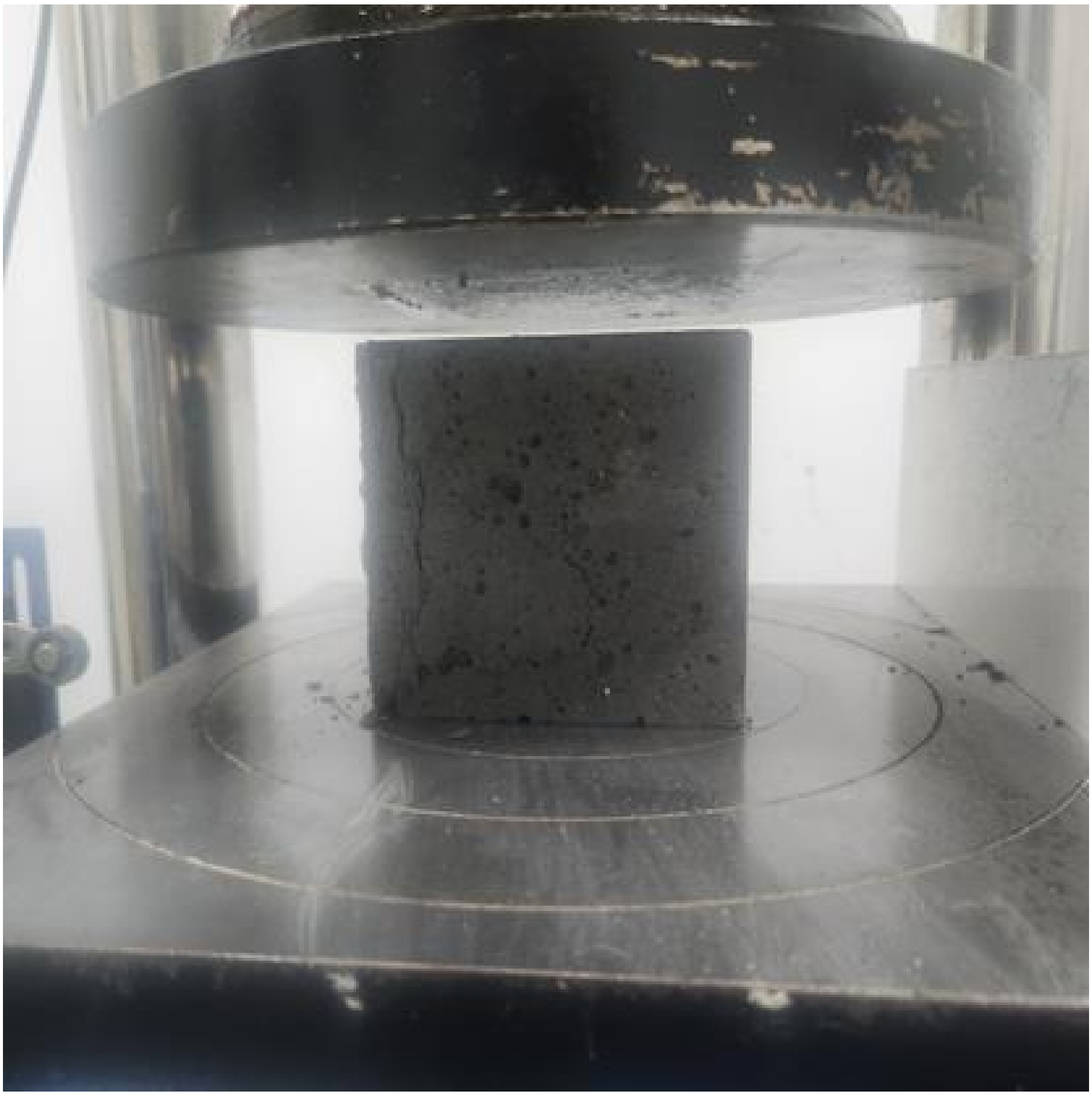

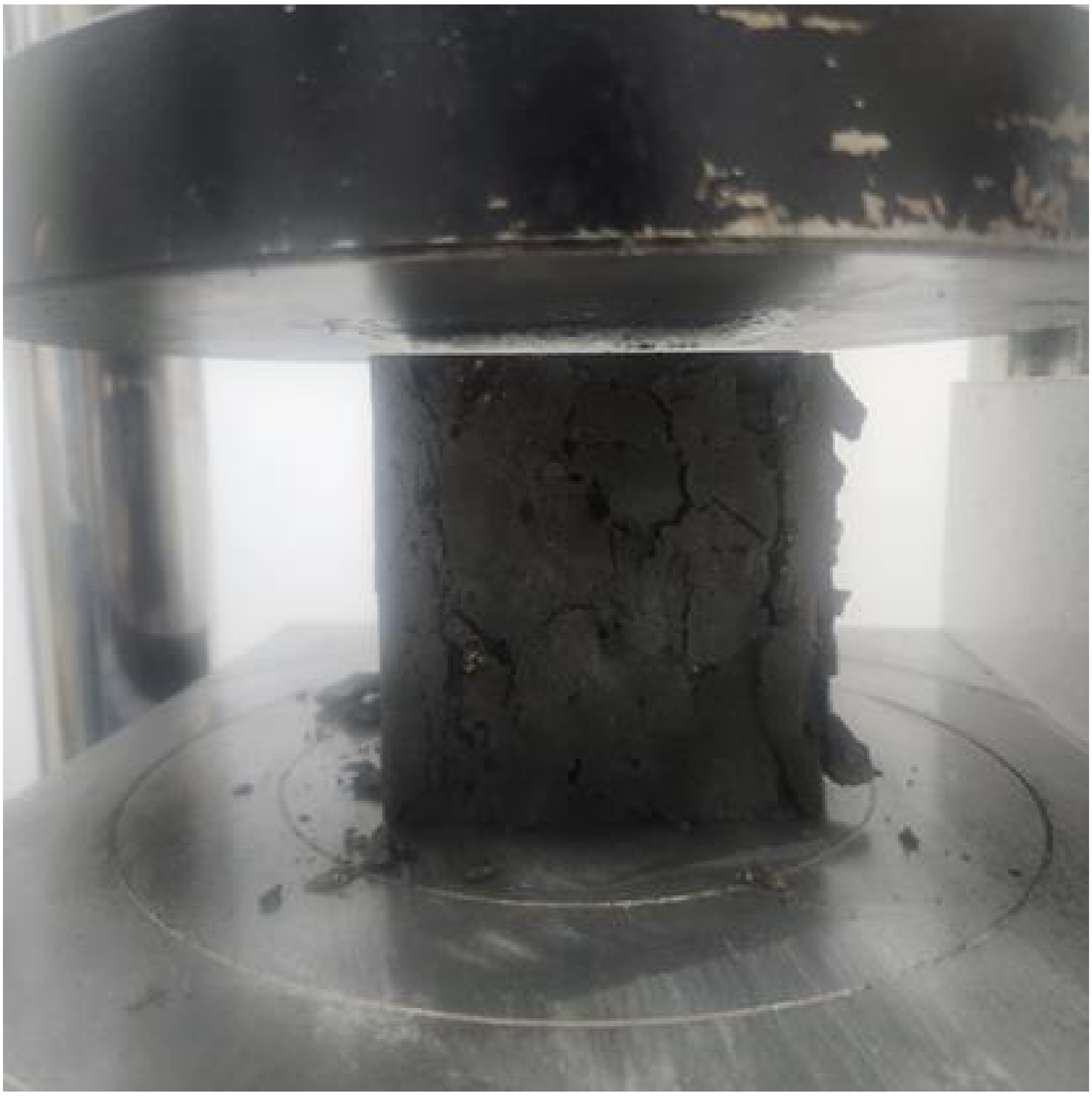

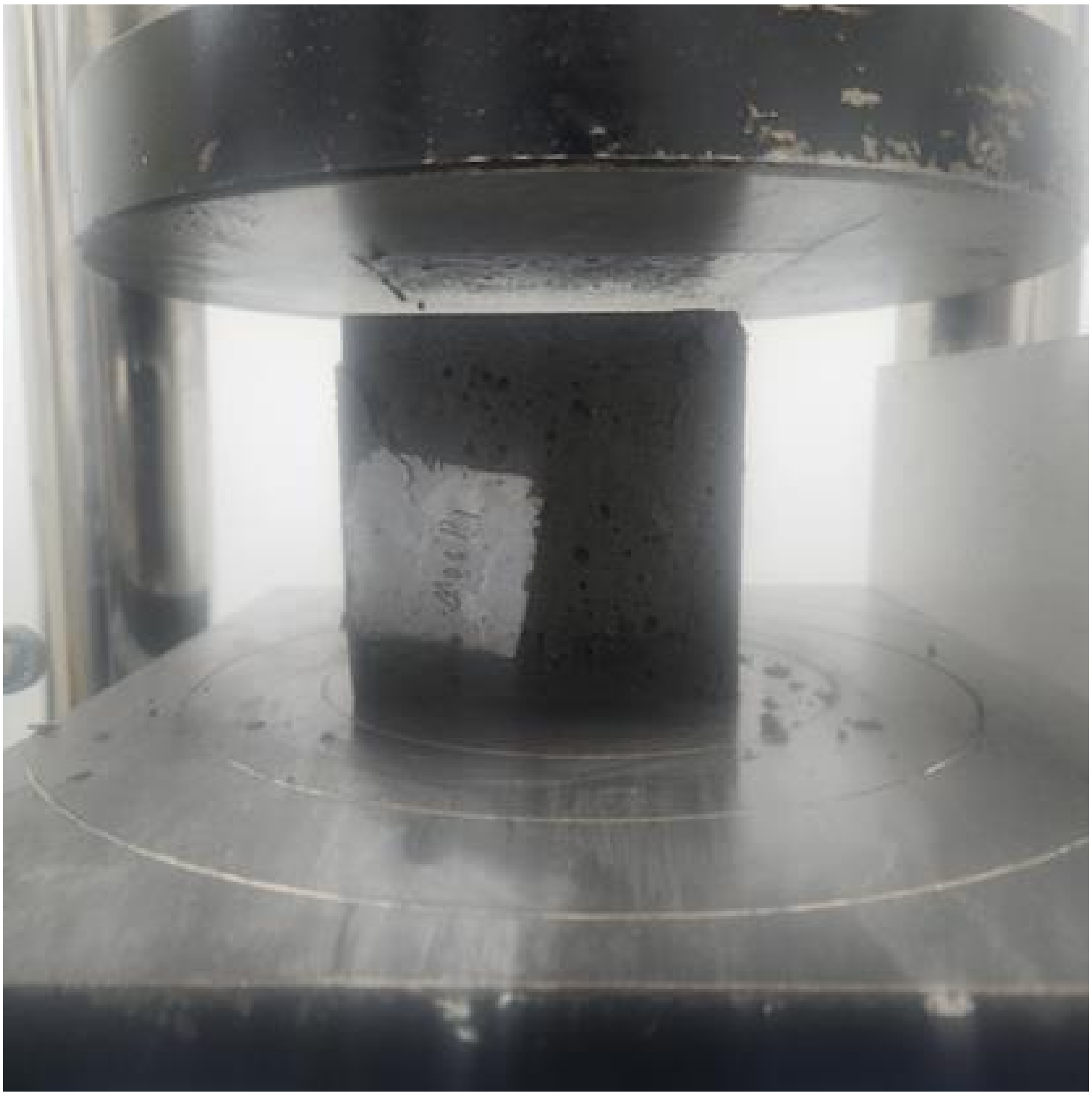

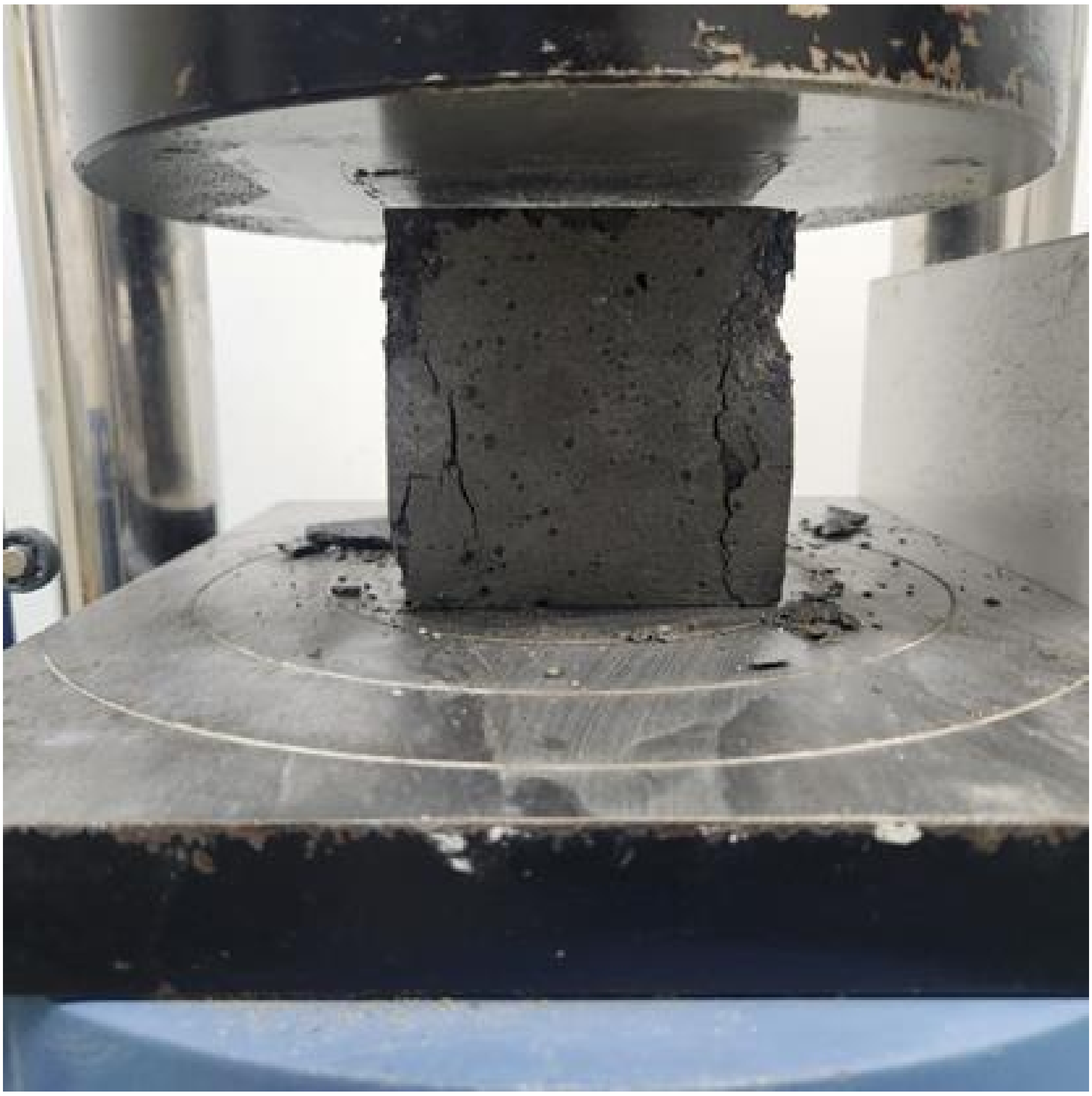

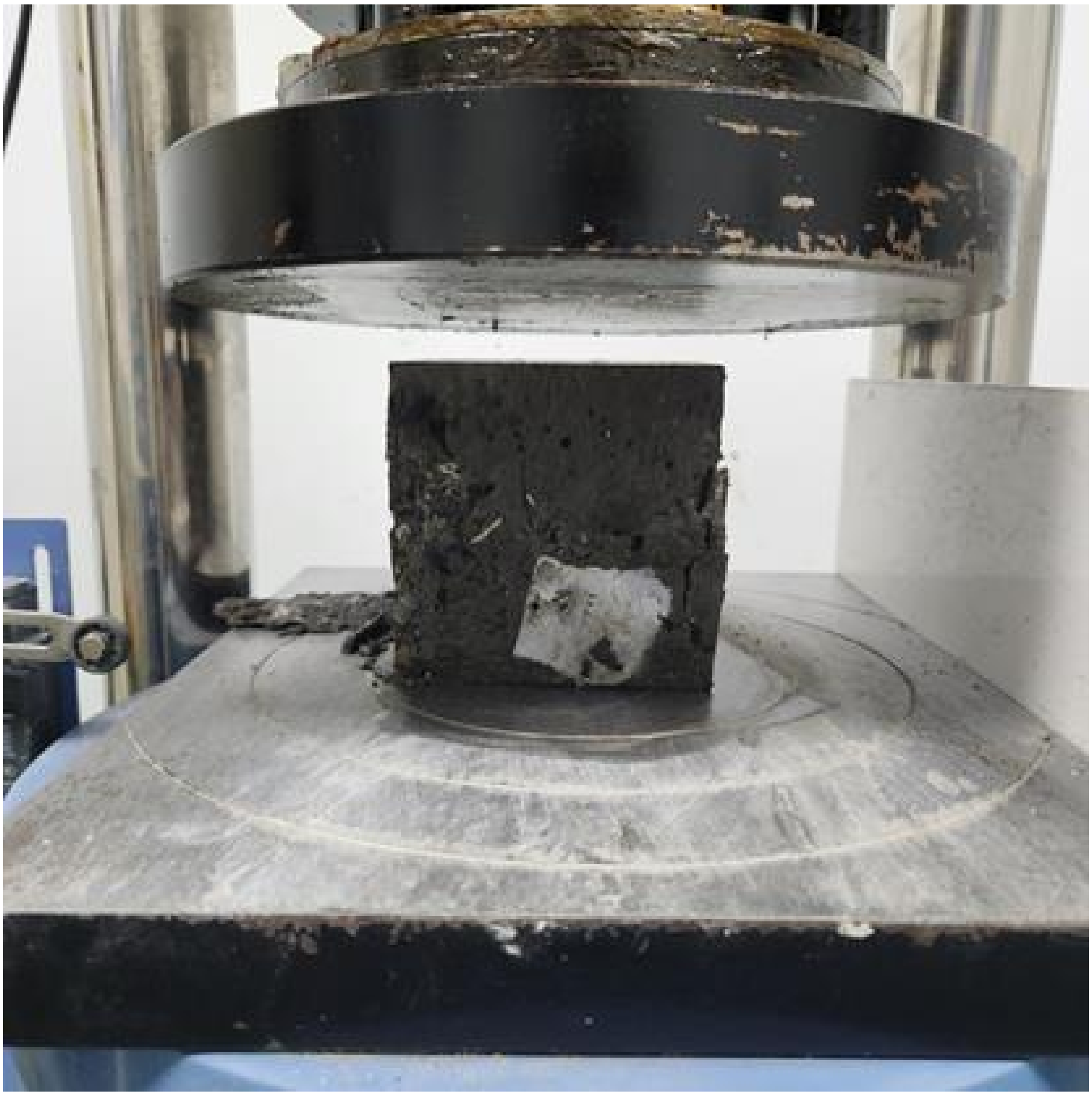

Supplement: S1 File — (PDF) [file pone.0331951.s001.pdf]
