# Supplementary material for: Research on mechanical properties and prediction methods of hybrid fiber concrete for airport pavements
Source: PLoS One. 2025 Nov 11;20(11):e0331951. doi: 10.1371/journal.pone.0331951 (PMC12604807; doi:10.1371/journal.pone.0331951)
Supplement: S2 File — (PDF) [file pone.0331951.s002.pdf]

S2 Image of data fitting process

```python
def calculate_y(a, b, c, B_j, phi_j, p_p, p_h):
    """
     y = a * b * c * (sum(B_j * phi_j)) * ((p_p / p_h) ** m) 的值

    :
    a -- a
    b -- b
    c --  c
    B_j --
    phi_j --
    p_p --

    :
    y --
    """
    m = 0.2  #
    term1 = a * b * c
    term2 = sum(B_j[j] * phi_j[j] for j in range(len(B_j)))
    term3 = (p_p / p_h) ** m

    y = term1 * term2 * term3
    return y

#
a = 1.03
b = 1.03
c = 0.97
B_j = [1.05, 1.1, 1.15]
phi_j = [0.3, 0.4, 0.3]
p_p = 100
p_h = 90

#
y = calculate_y(a, b, c, B_j, phi_j, p_p, p_h)
print("y =", y)
```

```python
#
def model(x, A, B, w, c):
    f0 = 49.2
    return f0 * (A + B * np.exp(-np.exp(-(x-c)/w) - (x-c)/w + 1))

#
x_data = np.array([26.3, 55, 45, 55])
y_data = np.array([58.32, 56.88, 56.22, 57.23])

# 给定的参数
A = 1.187350839516153
B = -0.044679690390025596
w = 10.413613222032385
c = 44.75995893081745
```
