# Supplementary material for: Research on mechanical properties and prediction methods of hybrid fiber concrete for airport pavements
Source: PLoS One. 2025 Nov 11;20(11):e0331951. doi: 10.1371/journal.pone.0331951 (PMC12604807; doi:10.1371/journal.pone.0331951)
Supplement: S3 File — (PDF) [file pone.0331951.s003.pdf]

S3 Variance, standard deviation, data

0.25	0.33	53.2	0.266
0.5	0.22	56.3	0.2815
0.75	0.42	57.8	0.289
1	0.53	58.2	0.291

0.05	0.33	49.6	0.248
0.1	0.22	50.3	0.2515
0.15	0.42	51.2	0.256
0.2	0.53	52.3	0.2615

PP1	0.33	58.1	0.2905
BF0.1	0.22	53.2	0.266
PVA0.2	0.42	52.3	0.2615
PAN0.2	0.53	52.4	0.262

PC				49.2				
PPFRC	48.11	1.6	4	51.14	2.62	7	46.5	1.22
BFRC	52.3	1.24	5	50.2	1.93	8	48.3	1.11
PNCC	50.2	1.77	6	51.3	2.5	9	49.3	1.09

PP1	0.023	5.8	0.029
BF0.15	0.052	5.4	0.027
PVA0.2	0.032	5.33	0.02665
PAN0.2	0.043	5.27	0.02635

0.05	0.023	4.98
0.1	0.052	5.12
0.15	0.032	5.22
0.2	0.043	5.27
